# Psyllium Fiber Protects Against Colitis Via Activation of Bile Acid Sensor Farnesoid X Receptor

**DOI:** 10.1016/j.jcmgh.2023.02.007

**Published:** 2023-02-23

**Authors:** Alexis Bretin, Jun Zou, Beng San Yeoh, Vu L. Ngo, Shawn Winer, Daniel A. Winer, Lavanya Reddivari, Michael Pellizzon, William A. Walters, Andrew D. Patterson, Ruth Ley, Benoit Chassaing, Matam Vijay-Kumar, Andrew T. Gewirtz

**Affiliations:** 1 Center for Inflammation, Immunity and Infection, Institute for Biomedical Sciences, Georgia State University, Atlanta, Georgia; 2 University of Toledo Microbiome Consortium, Department of Physiology and Pharmacology, University of Toledo College of Medicine and Life Sciences, Toledo, Ohio; 3 Department of Laboratory Medicine and Pathobiology, University of Toronto, Toronto, Ontario, Canada; 4 Buck Institute for Research on Aging, Novato, California; 5 Department of Food Science, Purdue University, West Lafayette, Indiana; 6 Research Diets, Inc., New Brunswick, New Jersey; 7 Department of Microbiome Science, Max Planck Institute for Biology, Tübingen, Germany; 8 Department of Veterinary and Biomedical Sciences, The Pennsylvania State University, University Park, Pennsylvania; 9 INSERM U1016, Team “Mucosal Microbiota in Chronic Inflammatory Diseases,” CNRS UMR 8104, Université Paris Cité, Paris, France

**Keywords:** inflammatory bowel disease, dietary fiber

## Abstract

**Background & Aims:**

Fiber-rich foods promote health, but mechanisms by which they do so remain poorly defined. Screening fiber types, in mice, revealed psyllium had unique ability to ameliorate 2 chronic inflammatory states, namely, metabolic syndrome and colitis. We sought to determine the mechanism of action of the latter.

**Methods:**

Mice were fed grain-based chow, which is naturally rich in fiber or compositionally defined diets enriched with semi-purified fibers. Mice were studied basally and in models of chemical-induced and T-cell transfer colitis.

**Results:**

Relative to all diets tested, mice consuming psyllium-enriched compositionally defined diets were markedly protected against both dextran sulfate sodium- and T-cell transfer-induced colitis, as revealed by clinical-type, histopathologic, morphologic, and immunologic parameters. Such protection associated with stark basal changes in the gut microbiome but was independent of fermentation and, moreover, maintained in mice harboring a minimal microbiota (ie, Altered Schaedler Flora). Transcriptomic analysis revealed psyllium induced expression of genes mediating bile acids (BA) secretion, suggesting that psyllium’s known ability to bind BA might contribute to its ability to prevent colitis. As expected, psyllium resulted in elevated level of fecal BA, reflecting their removal from enterohepatic circulation but, in stark contrast to the BA sequestrant cholestyramine, increased serum BA levels. Moreover, the use of BA mimetics that activate the farnesoid X receptor (FXR), as well as the use of FXR-knockout mice, suggested that activation of FXR plays a central role in psyllium’s protection against colitis.

**Conclusions:**

Psyllium protects against colitis via altering BA metabolism resulting in activation of FXR, which suppresses pro-inflammatory signaling.

## Graphical abstract

**Figure:**
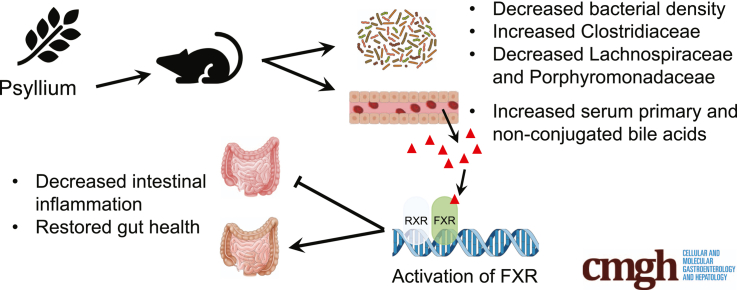

**Summary**

Psyllium protects against experimental colitis. Psyllium dramatically remodeled gut microbiota, but such changes were not critical for its anti-inflammatory action. Rather, psyllium increased serum bile acids, resulting in farnesoid X receptor activation, which mediated protection against dextran sulfate sodium-induced colitis.

Fiber has long been appreciated to be an important component of an optimal human diet that broadly promotes intestinal and metabolic health.^1^ In accord with this notion, reductionist animal models have found that enriching low-fiber diets with an array of specific semi-purified fibers provides protection against diet-induced metabolic syndrome.^2^ The extent of protection varies considerably for each fiber type with soluble fibers, which are readily fermented by gut bacteria, providing far greater protection than insoluble fibers, which are highly resistant to fermentation by gut bacteria.^3^ Mechanisms by which fermentable fibers protect against metabolic syndrome are thought to involve dampening of the low-grade inflammation that promotes this disorder.^4^, ^5^, ^6^ Such dampening of inflammation might reflect fiber-induced interleukin (IL)-22-mediated remediation of host-microbiota interactions and that fermentation products, namely short-chain fatty acids, promote development of regulatory T-cells potentially suppressing inflammation.^7^

In contrast, whether dietary fiber benefits more robust forms of intestinal inflammation, namely Crohn’s disease and ulcerative colitis, collectively referred to as inflammatory bowel disease (IBD), is less clear.^8^ In general, epidemiological studies suggest consumption of diets rich in fiber associate with reduced incidence of IBD, whereas some patients with IBD report inability to tolerate fiber-rich foods (eg, fermentable oligosaccharides, disaccharides, monosaccharides and polyols [FODMAPS]) and, moreover, blame fiber-rich foods for triggering acute flares of disease. In accord with the lack of consensus on the impact of dietary fiber on IBD, studies in mouse models of colitis report an array of distinct impacts by various specific semi-purified fibers, alleviating or exacerbating disease severity.^9^, ^10^, ^11^ Such impacts were frequently associated with distinct impacts on gut microbiota density and/or composition. Moreover, soluble/fermentable fibers such as inulin and pectin, which had beneficial impacts on host and microbial metabolism, particularly in ameliorating metabolic syndrome, generally exacerbated inflammation in murine colitis models.^10^^,^^11^ The notion that fermentable foods might negatively impact IBD has prompted many patients to consume low-fiber diets, thus missing out on the broad array of health benefits provided by fiber, which, at least for some fibers, may include an ability to dampen the inflammation that drives their disease. Hence, the goal of this study was to identify specific fibers that might ameliorate colitis. Moreover, we sought to understand the mechanism by which such a fiber(s) might suppress inflammation. We found that psyllium fiber strongly fulfilled our criteria but suppressed inflammation by a previously unrecognized mechanism, namely via activation of the bile acid nuclear receptor, farnesoid X receptor (FXR).

## Results

**Figure 1:**
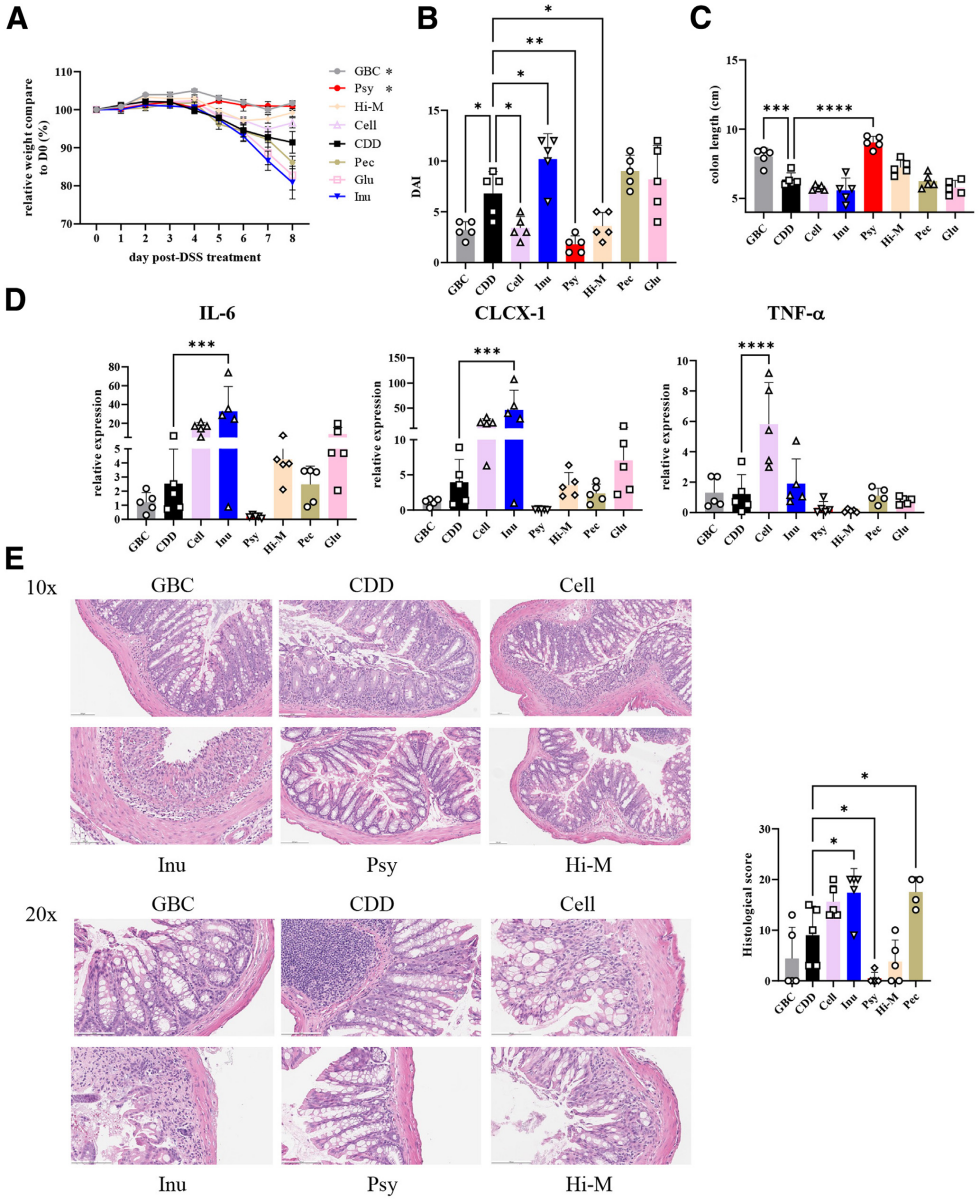
**Psyllium provides the strongest protection against DSS-induced colitis.** Male 6- to 8-week-old C57Bl/6 mice, which had been maintained on GBC, were administered the indicated diet for 7 days and subsequently challenged with drinking water containing DSS 2.5% for 8 days. (*A*) Relative body weight over time. (*B*) Disease activity index (DAI). (*C*) Colon length. (*D*) Relative expression of pro-inflammatory cytokines. (*E*) Histology score. Histology pictures are representative and are presented in 2 magnifications. Data are expressed as means ± SD of n = 5 mice per group. This experiment was performed 3 times, each showing a similar pattern of results. Significance was determined by analysis of variance test (all group are compared with the CDD group). ∗*P* < .05; ∗∗*P* < .01.

Mice used in biomedical research have historically been maintained on grain-based chow (GBC), which is made from unrefined heterogeneous seasonally variant ingredients that chemical analysis indicates contains 15% to 25% fiber, comprised of insoluble and soluble fiber, by weight.^12^ The high variability, and low manipulability, of GBC led diet-related research to rely on open-source compositionally defined diet (CDD) that are assembled from relatively invariant purified ingredients. CDD typically used as control diets contain only 5% fiber, all of which is insoluble fiber, namely cellulose. Consumption of CDD makes mice prone to colitis and metabolic syndrome.^13^ Enriching CDD with the soluble fiber inulin ameliorates metabolic syndrome but also confers high proneness to colitis, particularly in response to a chemical colitogen, dextran sulfate sodium (DSS), suggesting that, in some circumstances, this fiber can be detrimental.^3^^,^^10^^,^^11^ Hence, we examined impacts of other fibers, particularly soluble fibers, with the central goal of identifying a metabolism-benefiting fiber that would not have detrimental effects and would, in fact, protect mice against experimental colitis. Mice that had been raised on GBC were maintained on this diet or switched to CDD, or CDD enriched with 150 g of indicated semi-purified fiber, including psyllium, which is comprised of about 70% soluble fiber (Table 1).^14^ One week later, mice were challenged with DSS, and colitis severity was assessed by an array of clinical-type, morphologic immunopathologic, and histopathologic parameters (Table 2). In accord with previous studies, enriching CDD with inulin markedly exacerbated the severity of DSS colitis by all parameters assayed (Figure 1). Somewhat similar, albeit more moderate and variable, impacts were seen with cellulose, pectin, and glucomannan (Figure 1). In contrast, enrichment of CDD with the “functional fiber” Hi-maize 260 resistant starch, which is non-chemically modified corn starch based on high amylose corn, ameliorated severity of DSS colitis to that of mice fed GBC. Yet, greater and near complete protection against DSS colitis was observed in mice fed enriching CDD enriched with psyllium (Figure 1). Specifically, psyllium protected against DSS-induced colitis as assessed by weight loss (Figure 1*A*), disease activity index (Figure 1*B*), colon length (Figure 1*C*), proinflammatory cytokine levels (Figure 1*D*), and colon histopathologic analysis (Figure 1*E*). Screening of these fibers in a model of diet-induced obesity found that psyllium, but not Hi-maize, protected against obesity and its metabolic consequences.^15^ Considering these observations and that a previously published screen of other fiber types also found psyllium to be the most protective fiber tested in this colitis model,^9^ prompted us to better understand the breadth and mechanism of its protection against inflammation.

**Figure 2:**
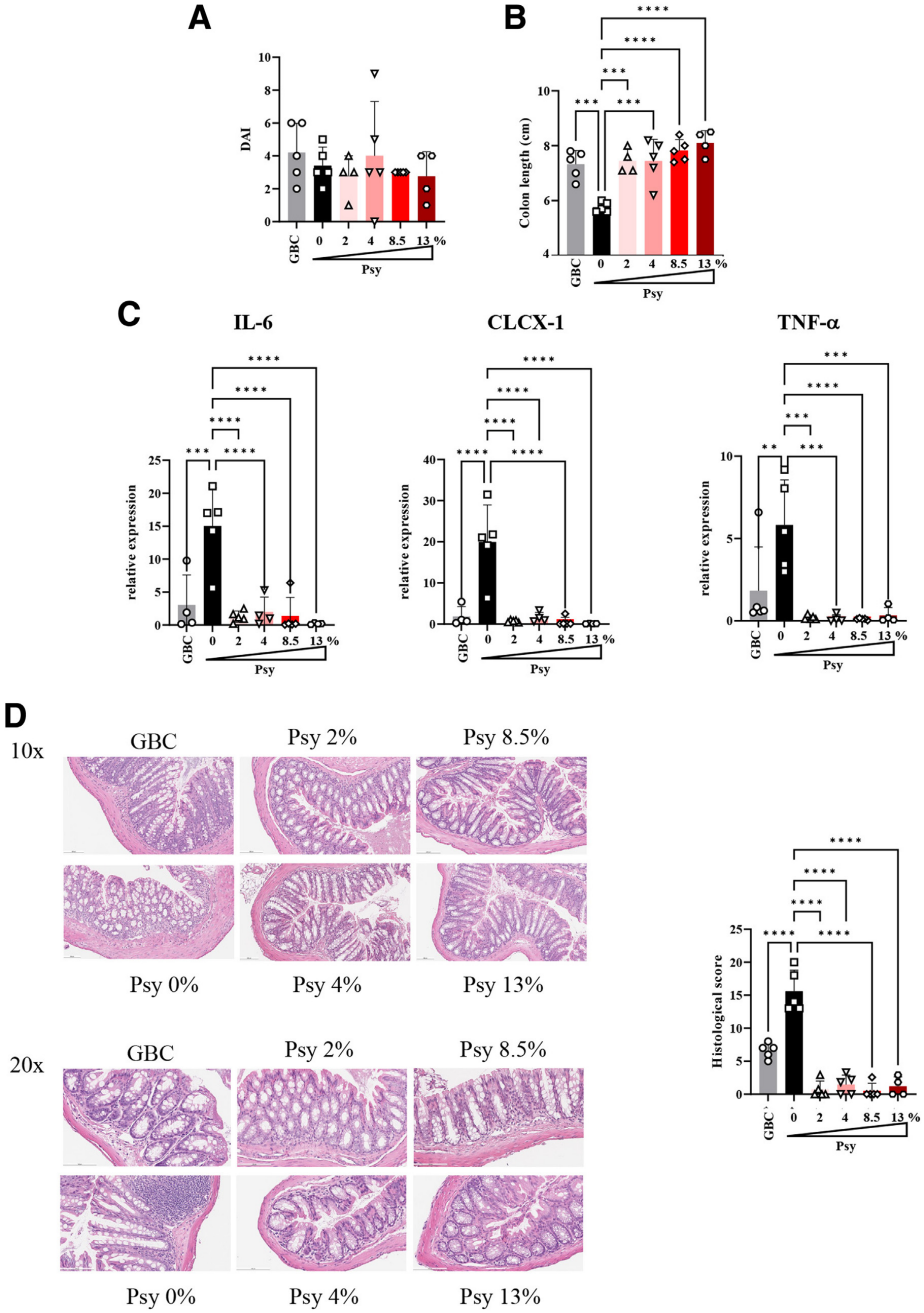
**Psyllium protected against DSS-induced colitis at lowest tested psyllium concentration 2%.** Male 6- to 8-week-old C57Bl/6 mice, which had been maintained on GBC, were administered the indicated diet for 7 days and subsequently challenged with drinking water containing DSS 2.5% for 8 days. (*A*) Disease activity index (DAI). (*B*) Colon length. (*C*) Relative expression of pro-inflammatory cytokines. (*D*) Histology score. Histology pictures are representative and are presented in 2 magnifications. Data are expressed as means ± SD of n = 5 mice per group. Significance was determined by analysis of variance test (all groups are compared with the 0% psyllium group). ∗∗*P* < .01; ∗∗∗*P* < .001; ∗∗∗∗*P* < .0001.

**Figure 3:**
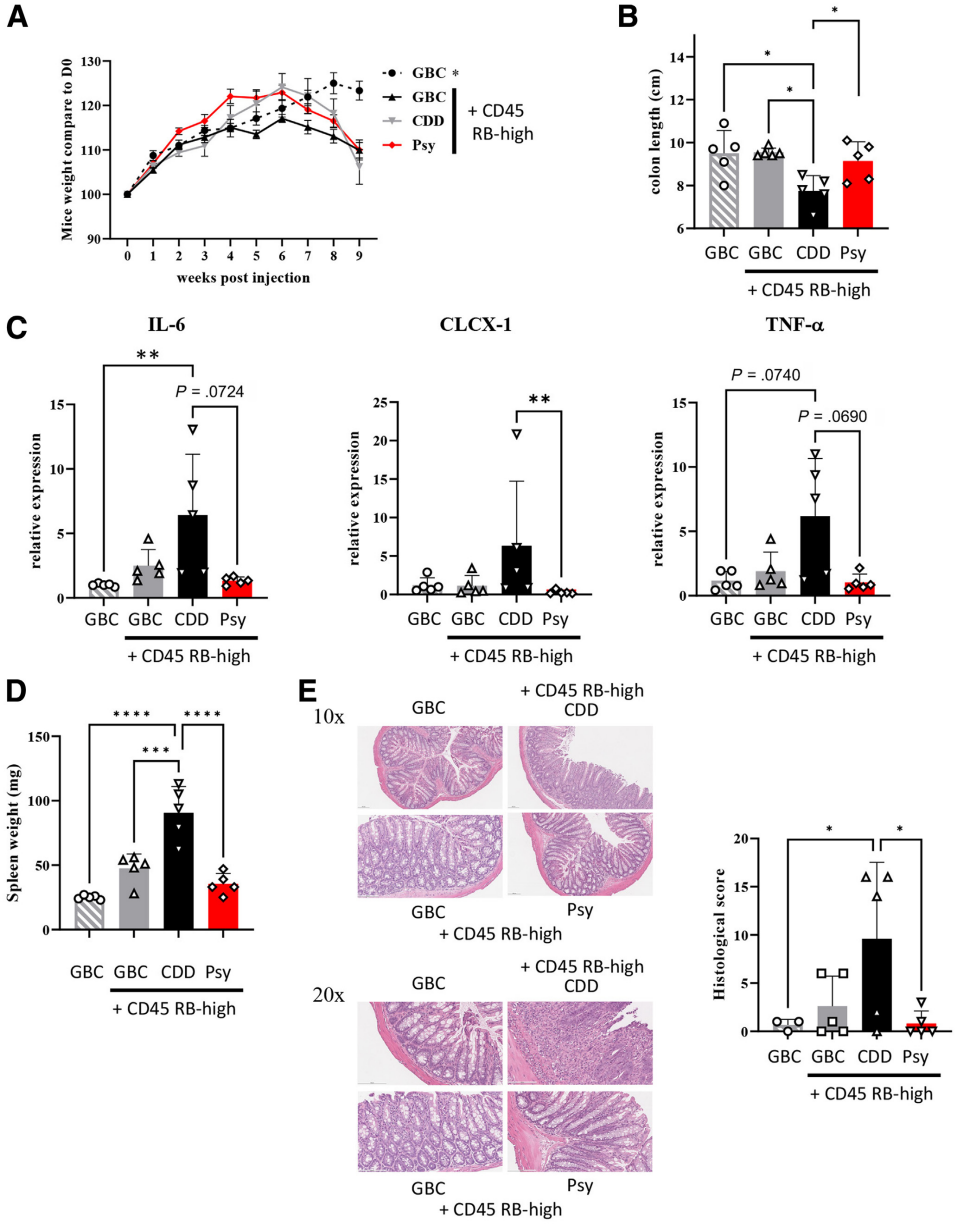
**Psyllium protected against colitis in the CD45 RB-hi transfer colitis model.** Rag1-knockout mice were maintained on the specified diet for 7 days and then injected with CD45 RB-hi cells and kept on the specified diet for 9 weeks. (*A*) Relative body weight over time. (*B*) Colon length. (*C*) Relative expression of pro-inflammatory cytokines. (*D*) Spleen weight. (*E*) Histology score. Histology pictures are representative and are presented in 2 magnifications. Data are expressed as means ± SD of n = 5 mice per group. This experiment was performed 2 times, each showing a similar pattern of results. Significance was determined by analysis of variance test (all group are compared with the CDD group) except for panel *C*, where a Kruskal-Willis test was performed. ∗*P* < .05; ∗∗*P* < .01; ∗∗∗*P* < .001; ∗∗∗∗*P* < .0001.

**Figure 4:**
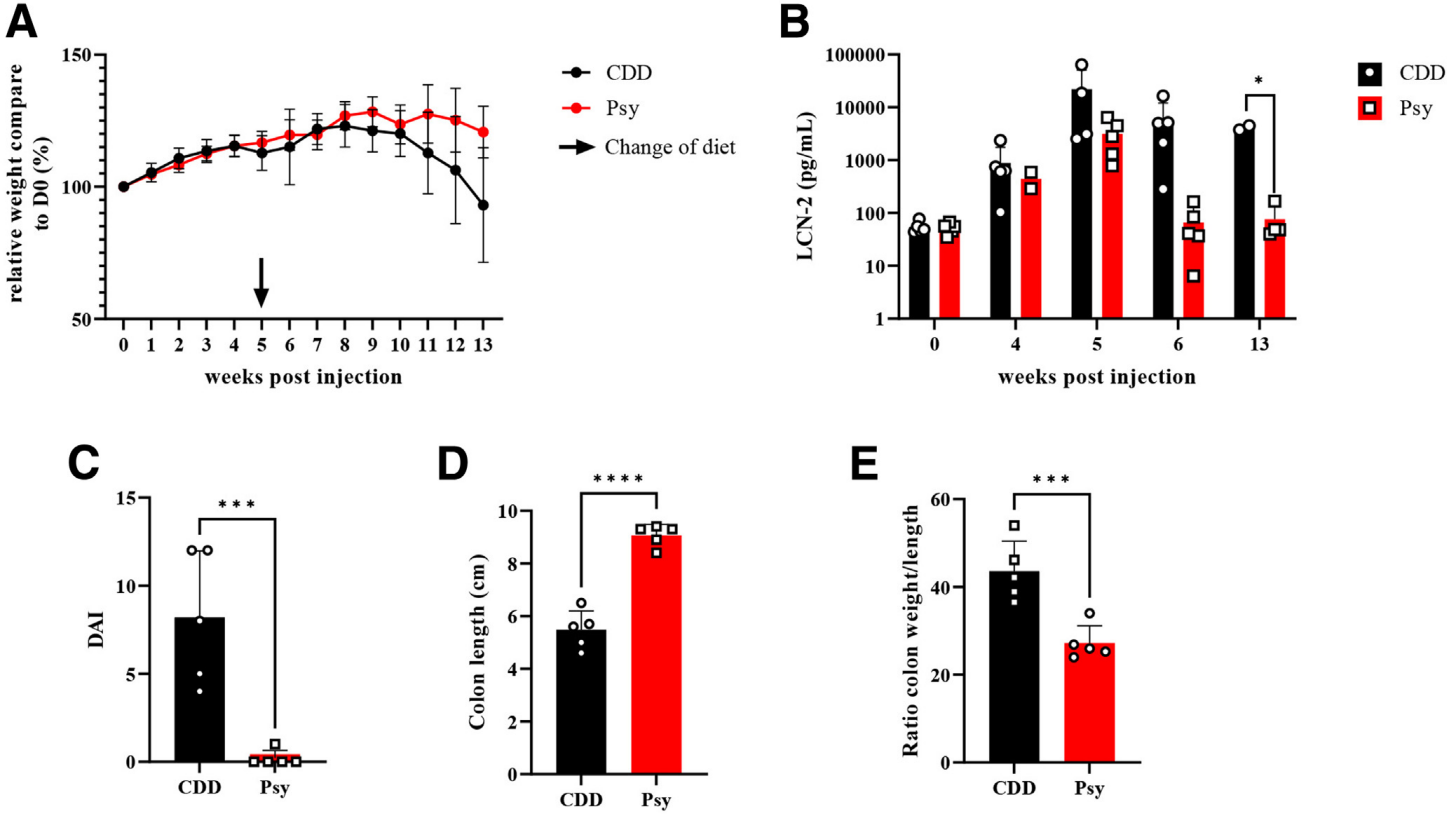
**Psyllium ameliorated colitis following disease onset in the CD45RB-hi transfer model.** Rag1 knockout mice were maintained on the specified diet for 7 days and then injected with CD45 RB-hi cells and maintained on GBC until they exhibited a clinical indication of disease and then switched to CDD and psyllium-enriched CDD. (*A*) Relative body weight over time. (*B*) LCN2 level in feces. (*C*) Disease activity index (DAI). (*D*) Colon length. (*E*) Ratio of colon weight/length. Data are expressed as means ± SD of n = 4 mice per group. Significance was determined by Student *t* test. ∗*P* < .05; ∗∗*P* < .01; ∗∗∗*P* < .001; ∗∗∗∗*P* < .0001.

We next examined the dose-dependence of psyllium’s protection against DSS colitis. We used diets with total approximate fiber content of 18% wt/wt, comprised of 0 to 130 gm/kg psyllium with the remaining fiber being cellulose. We found that diets comprised of as little as 2.0% (20 gm/kg) psyllium provided clear protection against DSS-induced colitis by all parameters assayed, whereas protection was most consistent across all parameters measured using diets containing 85 gm/kg psyllium (Figure 2). This dose of psyllium was also able to prevent the basal gut atrophy that results from CDD that lack fermentable fiber (Figure 2*B*) and, moreover was sufficient to protect against diet-induced metabolic syndrome.^15^ Thus, this dose was used to further study psyllium’s protection against colitis. That psyllium is reported to bind and remove xenobiotics from the intestine^16^ led us to consider the possibility that psyllium’s protection against DSS colitis might reflect its direct interaction with this chemical colitogen. However, the psyllium-enriched diet also provided strong protection in the T-cell mediated CD45RB^high^ colitis model. Specifically, relative to mice fed the matched CDD that lacked this fiber or relative to GBC-fed mice that had received the colitogenic T-cells (Figure 3), mice fed psyllium-enriched CDD exhibited reduced colitis severity as indicated by body weight (Figure 3*A*), colon length (Figure 3*B*), splenomegaly (Figure 3*D*), proinflammatory cytokine expression (Figure 3*C*), and histopathologic analysis (Figure 3*E*). We also used this colitis model to examine the extent to which a psyllium-enriched diet would ameliorate disease after a clinical-type indicator of its onset. Specifically, Rag1^-/-^ mice that had been administered colitogenic T-cells were maintained on GBC, which is standard practice with this model, until they exhibited a clinical indication of disease, namely weight loss (Figure 4*A*), which was accompanied by an increase in the inflammatory marker fecal Lcn-2 (Figure 4*B*). At this point, mice were switched to a control or psyllium-enriched CDD and monitored. Although the switch to CDD by itself appeared to result in a transient recovery, weight loss resumed a few weeks later, necessitating euthanasia, at which time mice exhibited severe colitis (Figure 4*C–E*). Such colitis was largely absent in mice fed CDD-psyllium. Thus, psyllium’s protection against inflammation is not specific to DSS and may be broadly applicable to preventing and treating colitis.

**Figure 5:**
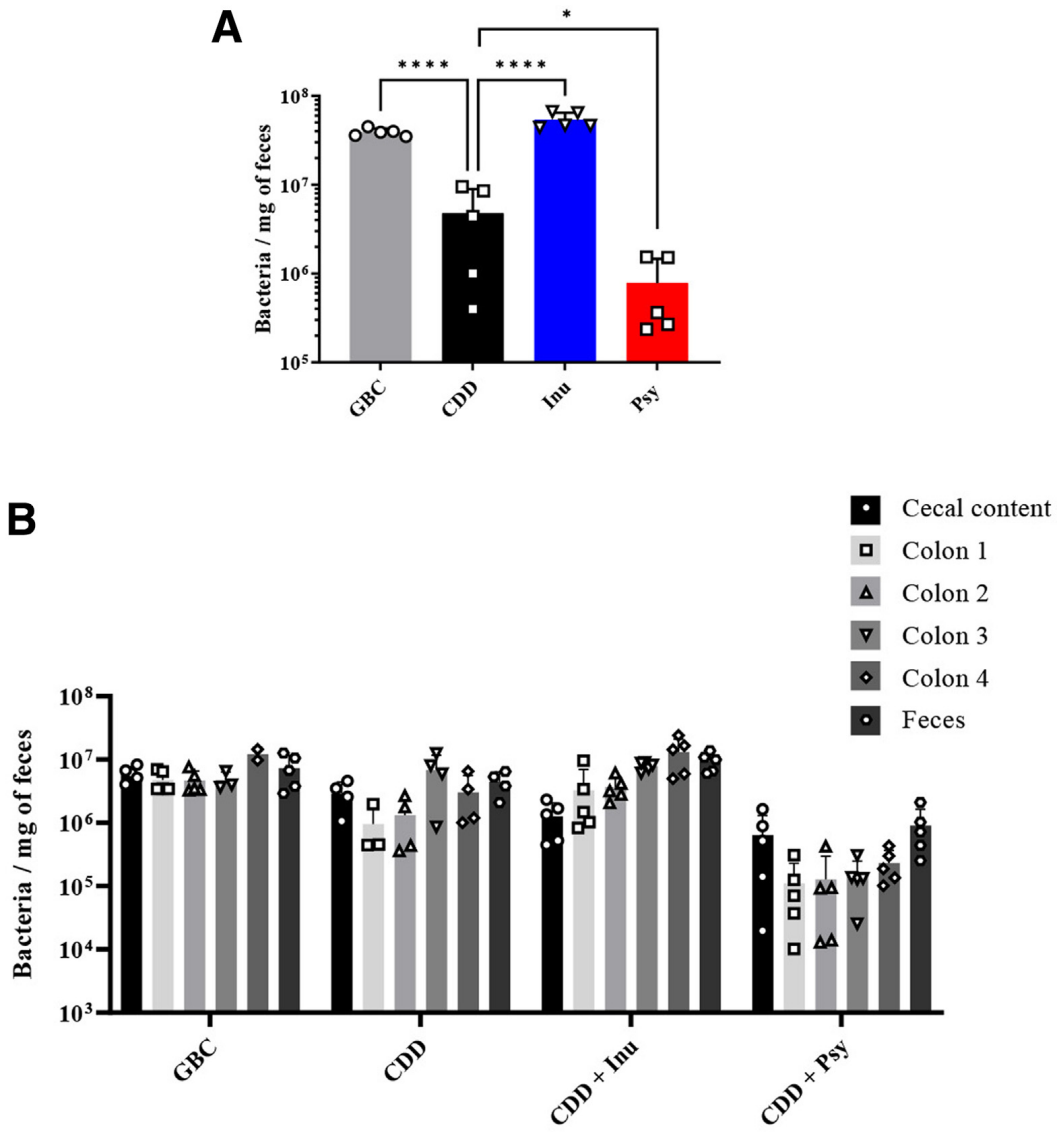
**Psyllium decreased bacterial density in the cecum, proximal, distal colon and feces.** Six- to eight-week-old C57Bl/6 male mice were maintained on the specified diet for 15 days, then feces and fecal matter from cecum, proximal colon, and distal colon were collected. (*A*) Bacterial density in the feces. Data are expressed as means ± SD of n = 5 mice per group and are representative of 3 independent experiments. Significance was determined by analysis of variance test (all groups are compared with the CDD group). (*B*) Bacterial density. Data are expressed as means ± SD of n = 4 mice per group. Significance was determined by analysis of variance test (multiple comparisons realized inside each group). ∗*P* < .05; ∗∗∗∗*P* < .0001.

**Figure 6:**
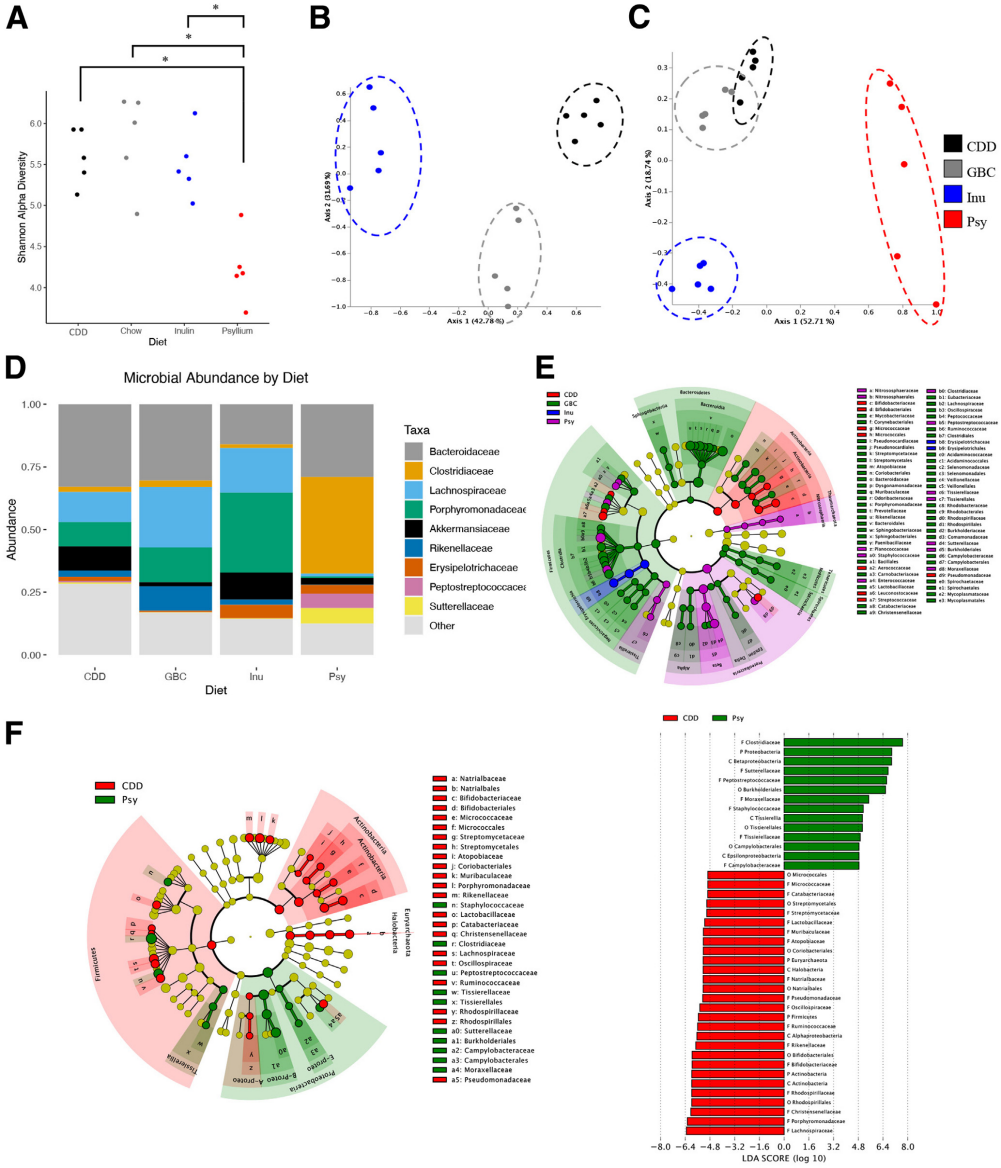
**Psyllium resulted in a unique microbiota composition.** Six- to eight-week-old C57Bl/6 male mice were maintained on the specified diet for 15 days and then feces were collected, DNA were extracted and sequenced. (*A*) Bacterial density. (*B*) Alpha diversity. (*C*) Beta diversity. (*D*) Taxonomy to the family level, with the 9 most abundant families shown and the remainder merged into Other. (*E*) Differences between the microbial taxonomic abundances of experimental groups tested with LEfSe’s linear discriminant analyses tests. The levels of taxonomy in the cladogram start at the domain level in the center, and indicate deeper levels towards the periphery (phylum, class, order, and the family level). *Green circles* indicate increased abundances in psyllium diets, *red* indicate increased abundance in CDD diet. Taxonomies detected as significant are shown alongside LDA values and are preceded by a P (phylum), C (class), O (order), or F (family). Significance was determined by analysis of variance test. ∗*P* < .05.

Dietary fiber is a major determinant of relative and absolute levels of gut bacteria, which can be envisaged to influence colitis severity in both DSS- and T-cell transfer-induced colitis models. In accord with previously observations, switching mice from GBC to CDD results in a rapid approximately 10-fold reduction in fecal gut bacterial density (Figure 5*A*).^3^ In further accord with our previous work, enriching CDD with inulin fully restored bacterial density but resulted in a starkly different microbiota composition, as evidenced by principal coordinate analysis, wherein we observed stark diet-based clustering between mice fed GBC, CCD, and inulin-enriched CDD (^3^ and Figure 6*B*). In striking contrast to inulin, enriching CDD with psyllium resulted in a further 5-fold reduction in bacteria per mg of feces (Figure 5*A*). This reduction in bacterial density was evident in the cecum and proximal and distal colon (Figure 5*B*). In accord with previous observations in germfree mice,^17^ psyllium’s lowering of microbiota associated with enlarged ceca (consumption of psyllium-enriched diet resulted in a 320% ± 20% increase in cecal mass; *P* < .0001). Psyllium also had strong impacts on gut microbiota composition, the extent of which dwarfed the substantial differences in mice fed CDD vs GBC (Figure 6*C*). Consequently, principal coordinate analysis of gut microbiota of mice fed GBC, CDD, and CDD enriched with inulin showed clear clustering of all 3 groups (Figure 6*B*), including samples from psyllium-fed mice in the analysis showed that differences along PC1 solely reflected impacts of psyllium, whereas impacts of CDD were no longer evident (Figure 6*C*). Psyllium’s impact on gut microbiota composition reflected a loss of α-diversity (Figure 6*A*) driven, in part, by loss of several families of Firmicutes, with some increases in Proteobacteria also being evident (Figure 6*D–F*). Furthermore, we note that psyllium seemed to restore clostridia and proteobacteria to levels similar to GBC (Figure 6*E*).

**Figure 7:**
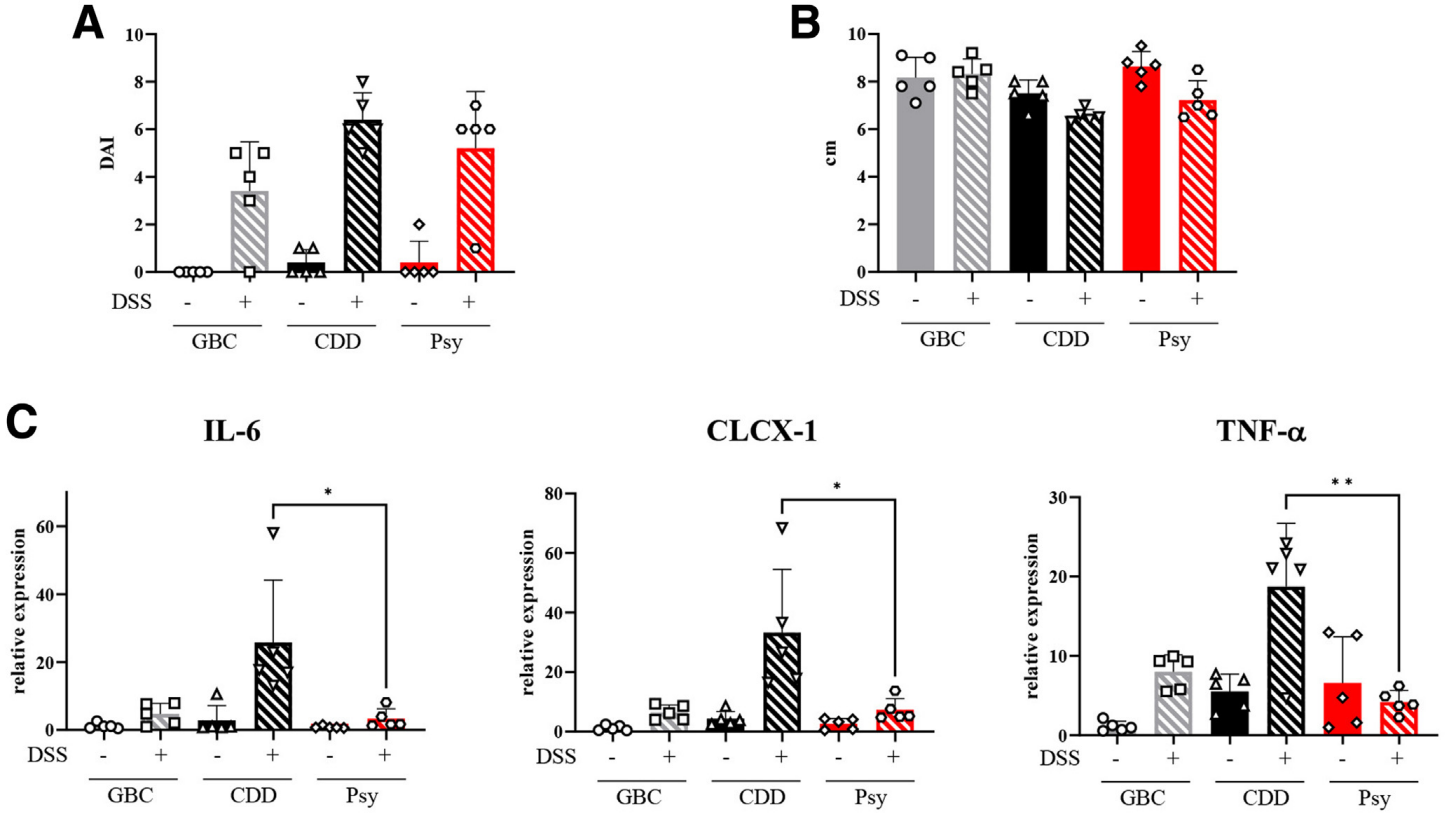
**Psyllium did not require fermentation to protect against DSS-induced colitis.** C57Bl/6 male mice were given b-acids in the water and maintained on the specified diet for 7 days and subsequently treated with DSS 2.5% for 8 days. (*A*) Disease activity index (DAI). (*B*) Colon length. (*C*) Relative expression of pro-inflammatory cytokines. Data are expressed as means ± SD of n = 4 mice per group. Significance was determined by analysis of variance test (all groups are compared with the CDD group). ∗*P* < .05; ∗∗*P* < .01.

**Figure 8:**
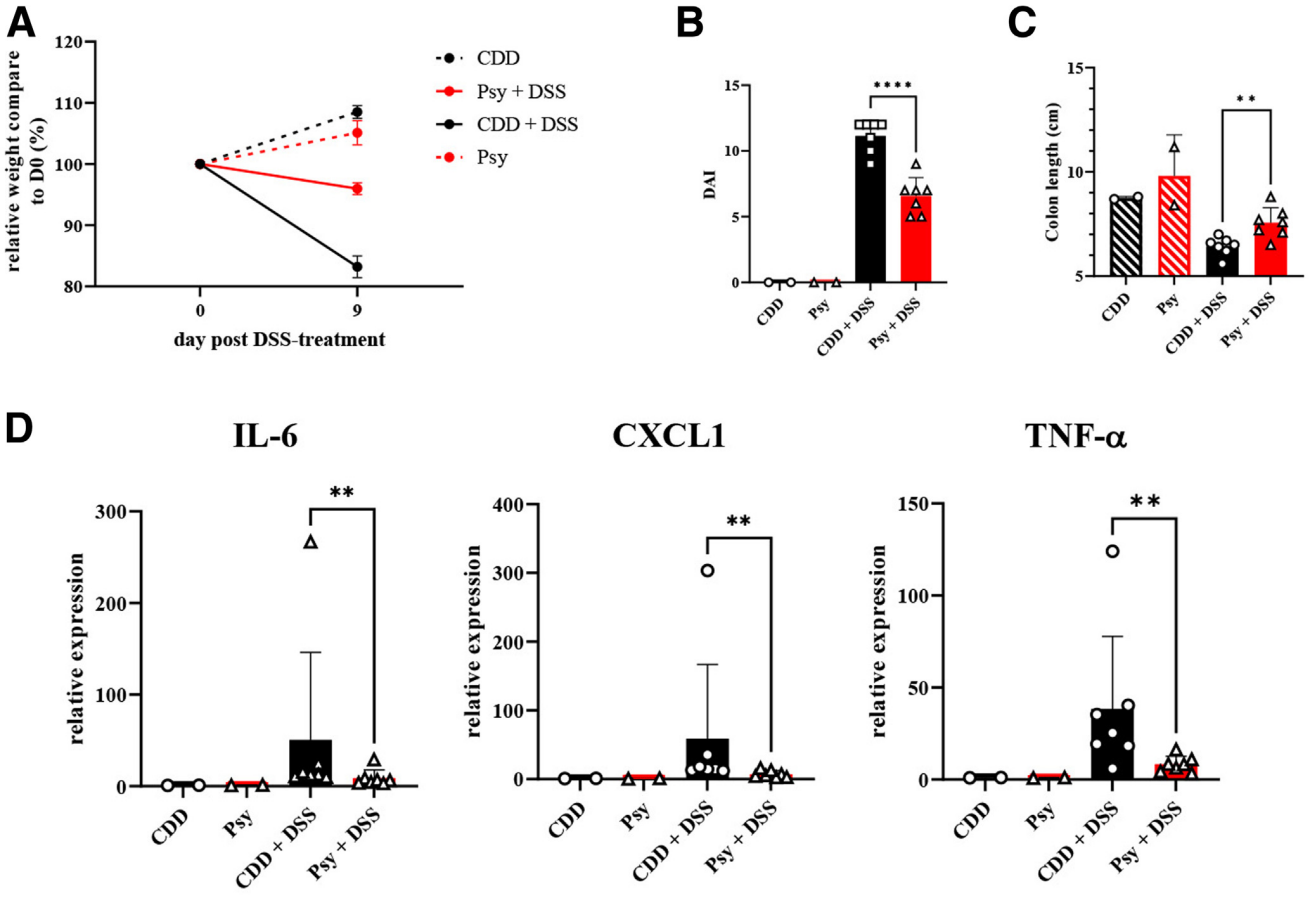
**Psyllium protected against DSS-induced colitis in ASF mice.** ASF male mice (C57Bl/6) were maintained on the specified diet for 7 days and subsequently treated with DSS 2.5% for 9 days. (*A*) Relative body weight over time. (*B*) Disease activity index (DAI). (*C*) Colon length. Data are expressed as means ± SD of n = 2 mice per groups without DSS and n = 7 mice per group with DSS. Significance was determined by analysis of variance test (all groups are compared with the CDD group). (*D*) Relative expression of pro-inflammatory cytokines. Data are expressed as means ± SD of n = 2 mice per groups without DSS and n = 7 mice per group with DSS. Significance was determined by Kruskal-Wallis test (all groups are compared with the CDD group). ∗*P* < .05; ∗∗*P* < .01; ∗∗∗∗*P* < .0001.

The stark reduction in bacterial density induced by psyllium argued against a role for IL-22 in mediating its protection against DSS colitis as expression of this cytokine is highly gut microbiota-dependent.^3^ Indeed, psyllium did not induce IL-22 expression (relative colonic mRNA IL-22 levels for mice fed CDD, CDD-inulin, and CDD-psyllium were 1.0 ± 0.6, 4.7 ± 0.3, and 0.14 ± 0.03, respectively; *P* = .0126). Psyllium’s reduction in gut microbiota density also argued against a role for fermentation, although in vitro studies indicate psyllium is semi-fermentable.^18^ Arguing against a role for fermentation, blocking fermentation with hops β-acids (added to drinking water at 20 ppm) did not impede psyllium’s protection against DSS colitis (Figure 7*A–C*). Although gut microbiota-dependence of a particular phenotype is often examined by use of germ-free mice, in the case of DSS colitis, germ-free status dramatically alters the course of DSS-induced disease, resulting in more gut necrosis than inflammation, thus making results difficult to interpret. Hence, we utilized mice with a minimal microbiota, namely gnotobiotic mice carrying only the Altered Schaedler Flora (ASF), which is an 8-species consortium that restores relatively normal gut metabolic and immune function.^19^ We observed that psyllium protected against DSS colitis in ASF mice, indicating that the complex changes in microbiota composition induced by this fiber are not absolutely required for protection against colitis (Figure 8), although the magnitude of protection, particularly regarding clinical type parameters and colon length was less striking than that observed in conventional mice, suggesting a portion of psyllium’s impacts in the DSS model may indeed require a complex microbiota. Psyllium continued to lower bacterial density in ASF mice (1.00 × 10^6^ ± 2.17 × 10^5^ vs 1.65 × 10^5^ ± 2.34 × 10^4^ bacteria/g of feces, respectively, for CDD- and psyllium-fed mice; *P* < .0001), leading to studies described below to investigate if this reduction in bacterial density contributed to its protection against DSS colitis.

**Figure 9:**
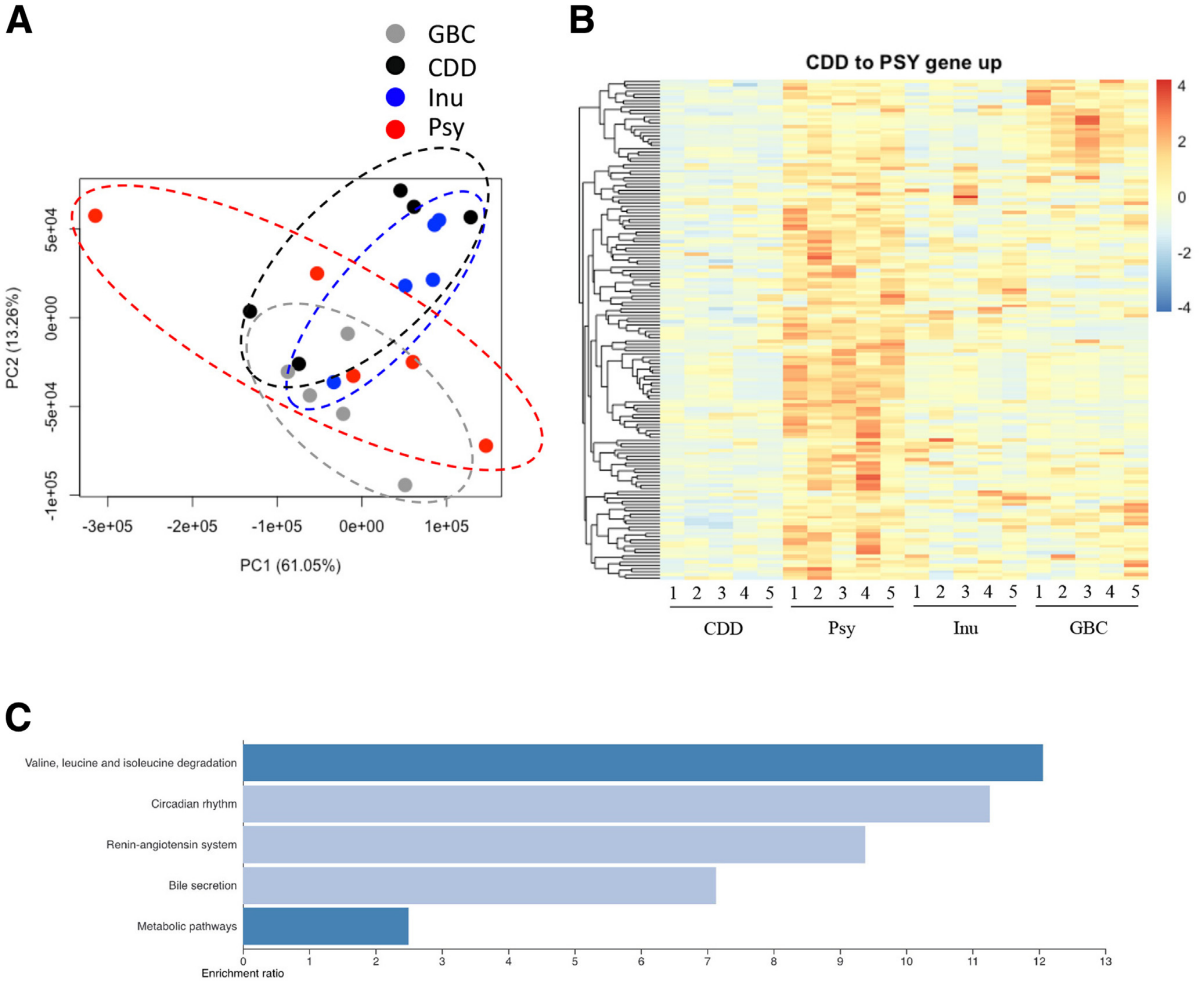
**Psyllium partially restored gene expression of GBC-fed mice and activated gene expression related to BA secretion.** Six- to eight-week-old C57Bl/6 male mice were maintained on the specified diet for 15 days and then colon samples were collected. RNA was extracted and sequenced. (*A*) PCA of gene expression variance between diet. (*B*) Heatmap of gene increase by 2fold between CDD and CDD enriched with psyllium. (*C*) Pathway represented by the gene increase by psyllium compared with CDD.

We next turned our attention to identifying host pathways that might mediate psyllium’s protection against inflammation, which we observed in models of both colitis and metabolic syndrome.^15^ We utilized a non-targeted approach, namely transcriptomic profiling, to broadly define how enriching CDD with psyllium impacts colon gene expression. These studies were done in the absence of DSS to increase our chances of identifying changes that contributed to, rather than simply reflected, the reduced level of inflammation. Relative to mice fed GBC, mice fed CDD showed stark changes in gene expression (Figure 9*A*). Enriching CDD with psyllium partially restored gene expression towards that of GBC-fed mice (Figure 9*A–B*). In this course, psyllium impacted expression of genes from a number of functional categories (Figure 9*C*), including hydrophobic amino acid degradation, circadian rhythm, renin-angiotensin system, and bile acid (BA) secretion.

**Figure 10:**
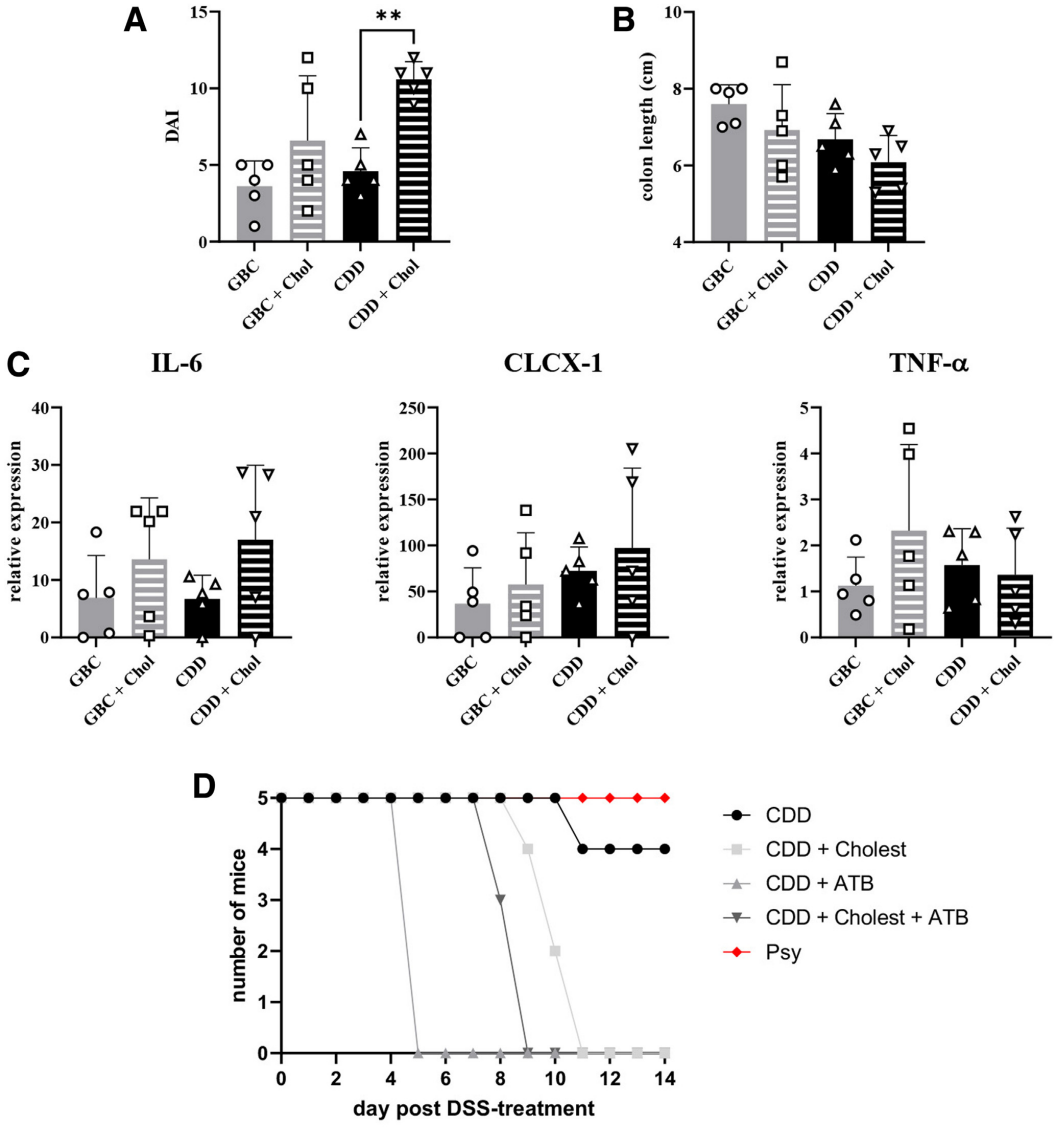
**Neither cholestyramine, antibiotics, nor their combination replicated psyllium’s protection against DSS-induced colitis.** (*A–C*) Six- to eight-week-old C57Bl/6 male mice were maintained on the specified diet for 7 days and subsequently treated with DSS 2.5% for 8 days in presence or absence of cholestyramine. (*A*) Disease activity index (DAI). (*B*) Colon length. (*C*) Relative expression of pro-inflammatory cytokines. Data are expressed as means ± SD of n = 5 mice per group. (*D*) Six- to eight-week-old C57Bl/6 male mice were maintained on the specified diet for 7 days and subsequently treated with DSS 2.5% for 14 days in presence or absence of cholestyramine and/or antibiotic. Survival curve. Data are expressed as means ± SD of n = 5 mice per group. Significance was determined by analysis of variance test (all groups are compared with the CDD group). ∗∗*P* < .01.

**Figure 11:**
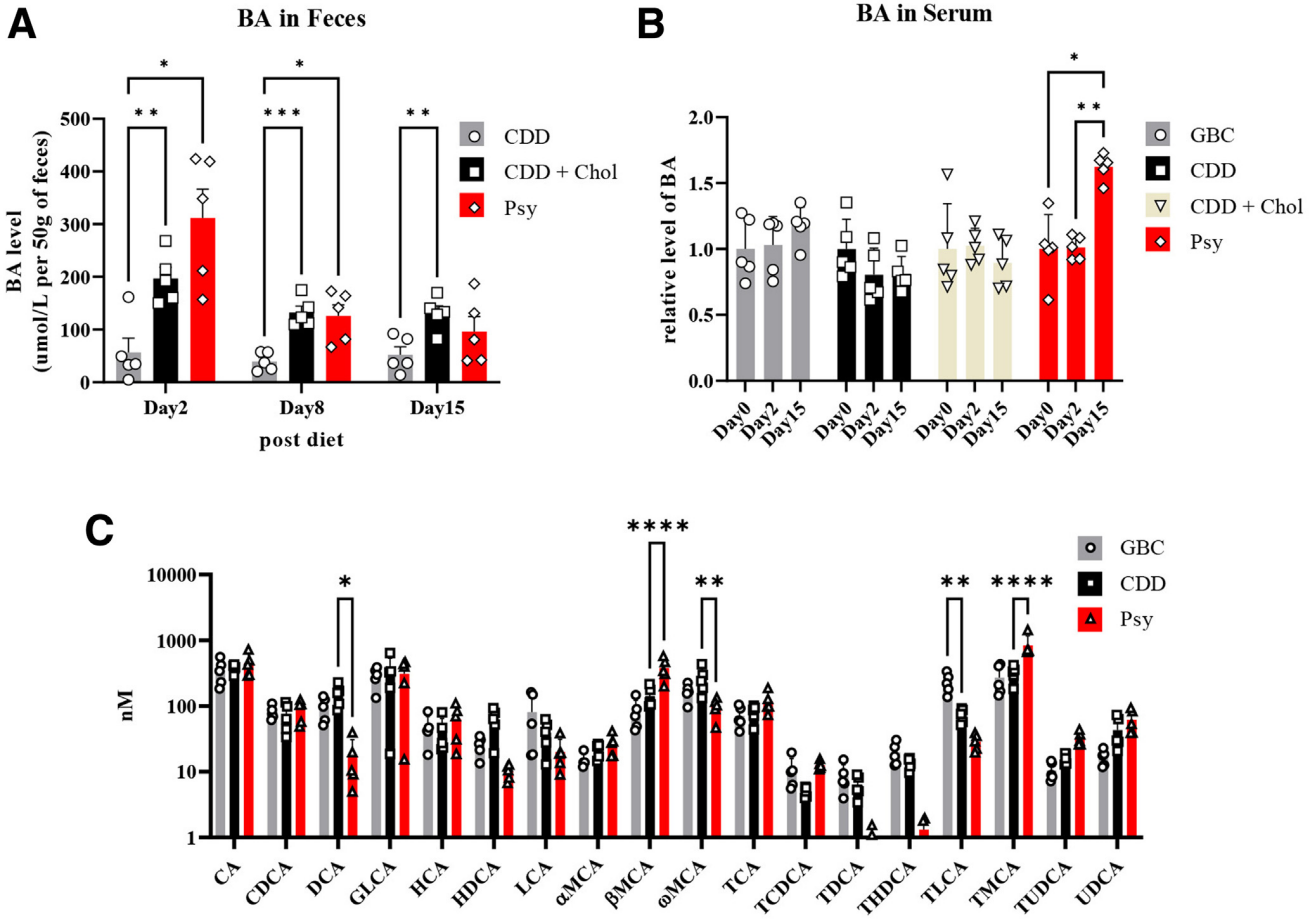
**Psyllium resulted in elevated BA in feces and serum.** C57Bl/6 male mice were maintained on the specified diet for 15 days, then serum and feces were collected. (*A*) Relative level of BA in the feces. (*B*) Relative level of BA in the serum. (*C*) Levels of BA species in the serum at day 15. Data are expressed as means ± SD of n = 5 mice per group. This experiment was performed 2 times, each showing a similar pattern of results. Significance was determined by analysis of variance test. ∗*P* < .05; ∗∗*P* < .01; ∗∗∗*P* < .001; ∗∗∗∗*P* < .0001.

The alteration of BA-related gene expression prompted us to consider if psyllium’s long-appreciated ability to bind BA and remove them from enterohepatic circulation, thus driving reverse cholesterol transport and lowering blood cholesterol, might also have contributed its suppression of colitis. To test this notion, we examined if the pharmacologic BA sequestrant, cholestyramine, might also impede DSS colitis. However, cholestyramine (added to diet at 2% by wt) did not protect against colitis and, in fact, appeared to exacerbate severity of DSS-induced colitis in mice fed GBC or CDD (Figure 10*A–C*). We also considered the possibility that psyllium’s reduction of gut bacterial density and BA chelation might work in concert to ameliorate colitis. However, combinations of cholestyramine and antibiotics, used at concentrations that lowered bacterial density to that of psyllium fed mice (aded to drinking water at following concentration: ampicillin (0.5 g/L), vancomycin (0.1 g/L), neomycin (0.5 g/L), and metronidazole (0.5 g/L), also failed to suppress DSS colitis (Figure 10*D*). These results argued against the notion that BA sequestration was contributing to psyllium’s prevention of DSS colitis. However, comparing impacts of cholestyramine and psyllium on BA assay of BA levels revealed distinct impacts of these products on BA metabolism. Specifically, as expected, both psyllium and cholestyramine led to elevated levels of fecal BA, reflecting their known abilities to sequester BA thus preventing their reabsorption (Figure 11*A*). The extent of elevated fecal BA peaked shortly after treatment initiation, likely reflecting inherent limits in the extent to which BA synthesis can be upregulated to replenish lost BA. However, although serum BA remained nearly constant in mice fed GBC, CDD, or mice administered cholestyramine, mice fed psyllium-enriched CDD displayed an increase in total serum BA (Figure 11*B*). Analysis of BA species profiles indicated that the upregulation was broad-based across primary BA, suggesting reduced metabolism of BA by gut microbiota (Figure 11*C* and Table 3). The increase in serum BA led us to hypothesize that psyllium might activate signaling through BA receptors, particularly FXR, which has been observed to impact severity of colitis.^20^

**Figure 12:**
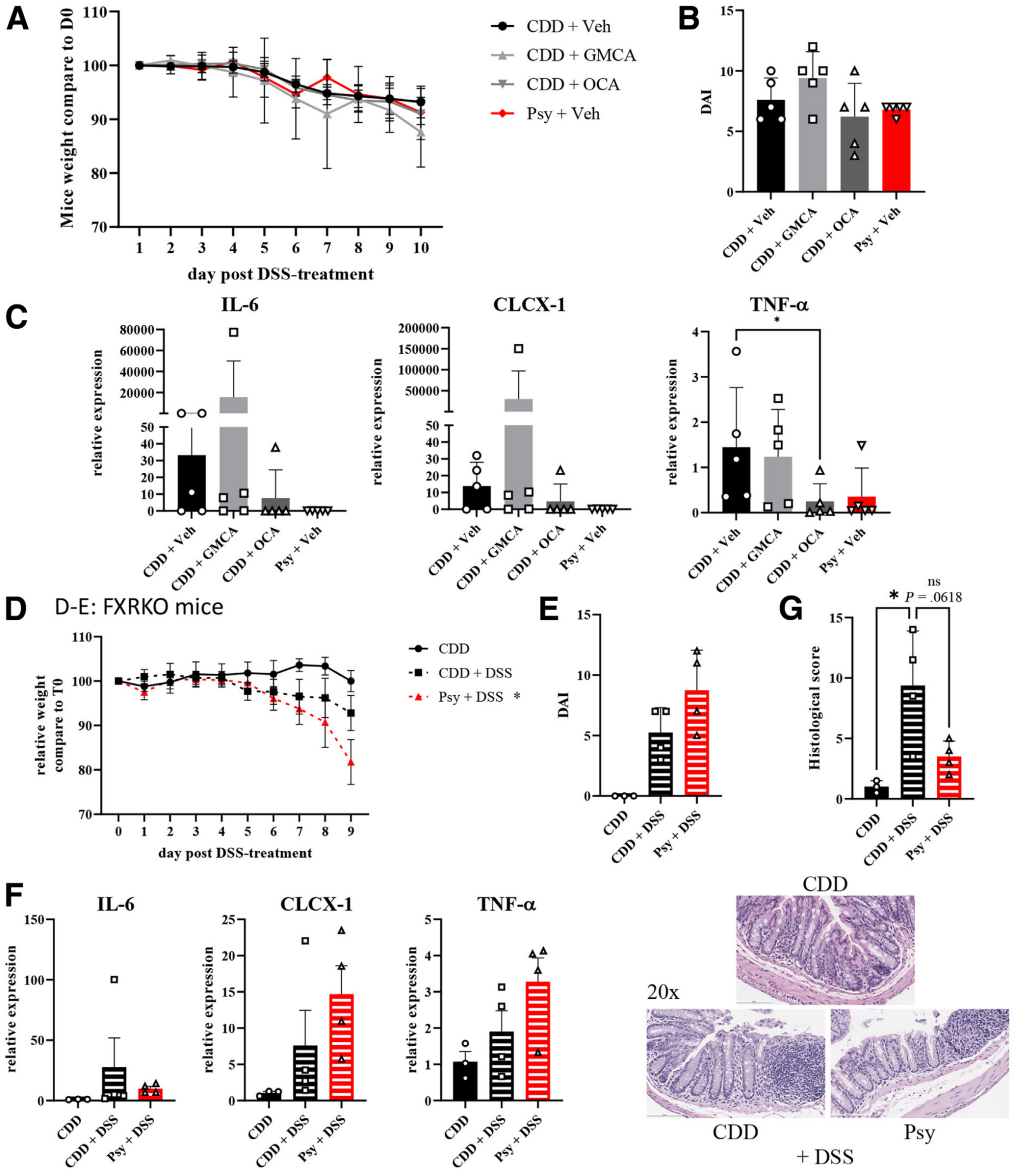
**Activation of FXR recapitulated and was necessary for psyllium’s protection against DSS-induced colitis.** (*A–C*) Six- to eight-week-old C57Bl/6 male mice were maintained on the specified diet for 7 days and gavage daily with vehicle (veh), FXR antagonist glyco-b-muricholic acid (GMCA), or FXR agonist obeticholic acid (OCA) and subsequently treated with DSS 2.5% for 10 days. (*A*) Relative body weight over time. (*B*) Disease activity index (DAI). (*C*) Relative expression of pro-inflammatory cytokines. Data are expressed as means ± SD of n = 5 mice per group. (*D–F*) Six- to eight-week-old FXRKO male mice were maintained on the specified diet for 7 days and subsequently treated with DSS 2.5% for 9 days. (*D*) Relative body weight over time. (*E*) Disease activity index (DAI). (*F*) Relative expression of pro-inflammatory cytokines. (*G*) Histology score. Histology pictures are representative. Data are expressed as means ± SD of n = 3 groups for CDD and n = 4 groups with DSS mice per group. This experiment was performed 2 times, each showing a similar pattern of results. Significance was determined by analysis of variance test (all groups are compared with the CDD group). ∗*P* < .05.

We first tested the notion that psyllium’s protection against colitis might involve FXR by examining the extent to which a pharmacological FXR ligand could recapitulate psyllium’s dampening of DSS-induced colitis. Mice were administered phosphate buffered saline, FXR agonist obeticholic acid, or FXR antagonist glyco-β-muricholic acid daily by oral gavage (20mg/kg diluted in PEG).^21^ The daily oral gavage, by itself, seemed to induce stress that interfered with our measures of clinical-type parameters (Figure 12*A–B*). Nonetheless, assessing the extent of DSS-induced inflammation via measure of pro-inflammatory cytokine expression indicated that, like psyllium, the FXR agonist markedly ameliorated colitis severity (Figure 12*C*). In contrast, the FXR antagonist glyco-β-muricholic acid potentiated DSS-induced inflammation (Figure 12*A–C*). We next examined a potential role for FXR in mediating psyllium’s protection against DSS colitis via use of FXR-deficient mice. Analogous to wild-type (WT) mice, FXR-deficient mice displayed increased serum BA in response to CDD enriched with psyllium (mean serum BA ± standard deviation [SD] was 12.8 ± 5.0 μmol/L and 51.8 ± 18.3 μmol/L, respectively, for mice fed with CDD and psyllium-enriched CDD; *P* = .0369). However, the ability of psyllium-enriched diet to protect mice against DSS-induced colitis was largely ablated. Rather, the psyllium-enriched diet appeared to modestly exacerbate the clinical-type disease manifestations and production of pro-inflammatory cytokines in FXR-deficient mice, although histopathologic scoring indicated a trend of mild protection, albeit not statistically significant, in the psyllium-fed mice was observed (Figure 11*D–F*). These results indicate that psyllium’s protection against colitis involves its ability to increase circulating BA levels, thus activating FXR signaling.

## Discussion

Dietary fiber has long been appreciated to be health-promoting, but the mechanisms by which it acts are not fully understood. Attempts to address this question have generally categorized fiber by its structural and consequently, biochemical properties, particularly factors that impact its solubility and thus the extent to which the complex carbohydrates that comprise each specific fiber are accessible to gut bacteria. Accordingly, impacts of soluble fibers, in humans and mice, are thought to be mediated by gut bacteria, whereas those of insoluble fibers have generally been attributed to their ability to provide bulk, impact motility, and/or bind noxious substances, thus leading to their removal. Such actions of insoluble fiber may explain the broad generally beneficial but modest impacts of cellulose in a variety of models of inflammatory diseases.^3^^,^^9^ Conversely, the pivotal and complex role of gut microbiota in these diseases may underlie why nourishing gut bacteria with some fermentable fibers alter microbiota in a manner that dampens low-grade inflammation that drives metabolic syndrome but exacerbates the acute inflammation observed in DSS colitis.^11^ Yet this logic holds that other fermentable fibers might have distinct impacts, with perhaps some fibers altering host-microbiota interactions in a manner that may be broadly beneficial prompting us to seek fiber(s) that could protect mice against diet-induced obesity and colitis.

Testing a panel of semi-purified fibers in mice led us to appreciate that psyllium, which is semi-soluble and derived from Plantago seeds, was unique in its ability to provide marked protection against obesity and colitis. Such protection contrasts with other soluble fibers (eg, pectin and inulin), which protect against diet-induced obesity but can exacerbate experimental colitis.^10^^,^^11^ This observation prompted our focus on investigating psyllium’s mechanism of action focusing on its unique attribute, namely its ability to ameliorate colitis. Like other soluble fibers, psyllium starkly impacted gut microbiota composition. In fact, impacts of psyllium on gut microbiota composition dwarfed that of other fibers. However, in contrast to other soluble fibers that increase absolute abundance of bacteria (ie, fecal/luminal bacterial density), psyllium markedly decreased this parameter. Yet our data did not formally demonstrate a role for psyllium’s impact on gut microbiota in mediating its protection against colitis. Rather, that protection was maintained in mice with minimal microbiotas (ASF mice) and moreover, was not mimicked by antibiotics that similarly reduced bacterial density argued that the complex changes induced in the microbiota by psyllium are not absolutely necessary for protection. However, our observations do not rule out that changes in microbiota contribute to psyllium’s protection. First, psyllium did indeed impact the ASF microbiota. Additionally, the extent of protection observed in ASF mice appeared to be reduced relative to conventional mice, although we did not perform enough experiments to know if this difference would achieve statistical significance. Thus, overall, our results accord with findings and conclusions of Llewyn et al that psyllium protects against DSS colitis by both microbiota-dependent and microbiota-independent mechanisms.^9^ More generally, the ability of different dietary fibers to differentially alter microbiota might explain why some fermentable fibers, including inulin, exacerbate severity of DSS colitis.

Nonetheless, that a portion of psyllium’s protection against DSS colitis is microbiota-independent led us to consider that psyllium’s protection in this model might be related to this fiber’s long-recognized ability to impact BA metabolism.^22^ This led to the surprising finding that psyllium increased serum BA. Given the series of papers that show FXR activation protects against colitis in mouse models,^20^^,^^23^^,^^24^ we hypothesized that psyllium might protect against colitis via FXR activation, a notion supported by our finding that psyllium’s protection against DSS colitis was largely absent in FXR-deficient mice. More specifically, measure of clinical type disease indices, gross organ morphology and pro-inflammatory cytokines, indicated complete absence, and perhaps even reversal, of protection. Assessment of colitis by histopathologic scoring produced an equivocal result that suggested psyllium might continue to protect against tissue damage in the absence of FXR. Although such protection was not statistically significant, it nonetheless raises the possibility that FXR activation impedes pro-inflammatory immune responses, but yet psyllium may have another means of protecting the colon from damage. Further research will be needed to probe these possibilities.

How/why psyllium consumption elevates serum BA is not yet clear. We hypothesized that the increase in BA and intestinal BA transport gene expression was a compensatory response that enabled replacement of the enteric BA that were removed from enterohepatic circulation as a result of their being bound to dietary psyllium and excreted. However, that administration of cholestyramine led to similarly elevated levels of fecal BA did not alter levels of serum BA argues that BA sequestration alone might not explain psyllium’s elevation of serum BA. Moreover, a newly published study from Artis and colleagues indicates that inulin, which is not known to bind BA and which did not increase fecal BA, also increases serum BA.^25^ Use of gnotobiotic mice monoassociated with a single bacterial species showed that inulin-induced elevations in serum BA required bacterial bile salt hydrolase. Yet, given that impacts of psyllium and inulin on microbiota composition and density are starkly different, and that bile salt hydrolase is ubiquitous among gut bacteria, it is hard to envision these fibers sharing a microbiota-mediated mechanism that would account for increased serum BA. One potential cause of elevated serum BA is blockage of bile secretion (ie, cholestasis), resulting in BA accumulating in liver, spilling into systemic circulation, and potentially injuring the liver and other organs. That the levels of serum BA of psyllium-treated mice were well below those of cholemic mice, and mice maintained on psyllium-enriched diets for up to 6 months showed no overt health problems, argued against such mice having major blockage of BA secretion. Nonetheless, we cannot rule out the possibility that psyllium somehow modestly impedes BA secretion, although we are not aware of any reason why it would so. Thus, we speculate that psyllium but not cholestyramine increasing serum BA may reflect their have distinct affinities for select BA species and thus differentially impacting BA resorption and, consequently, synthesis, but at present, this scheme remains inchoate and is not backed by evidence.

**Figure 13:**
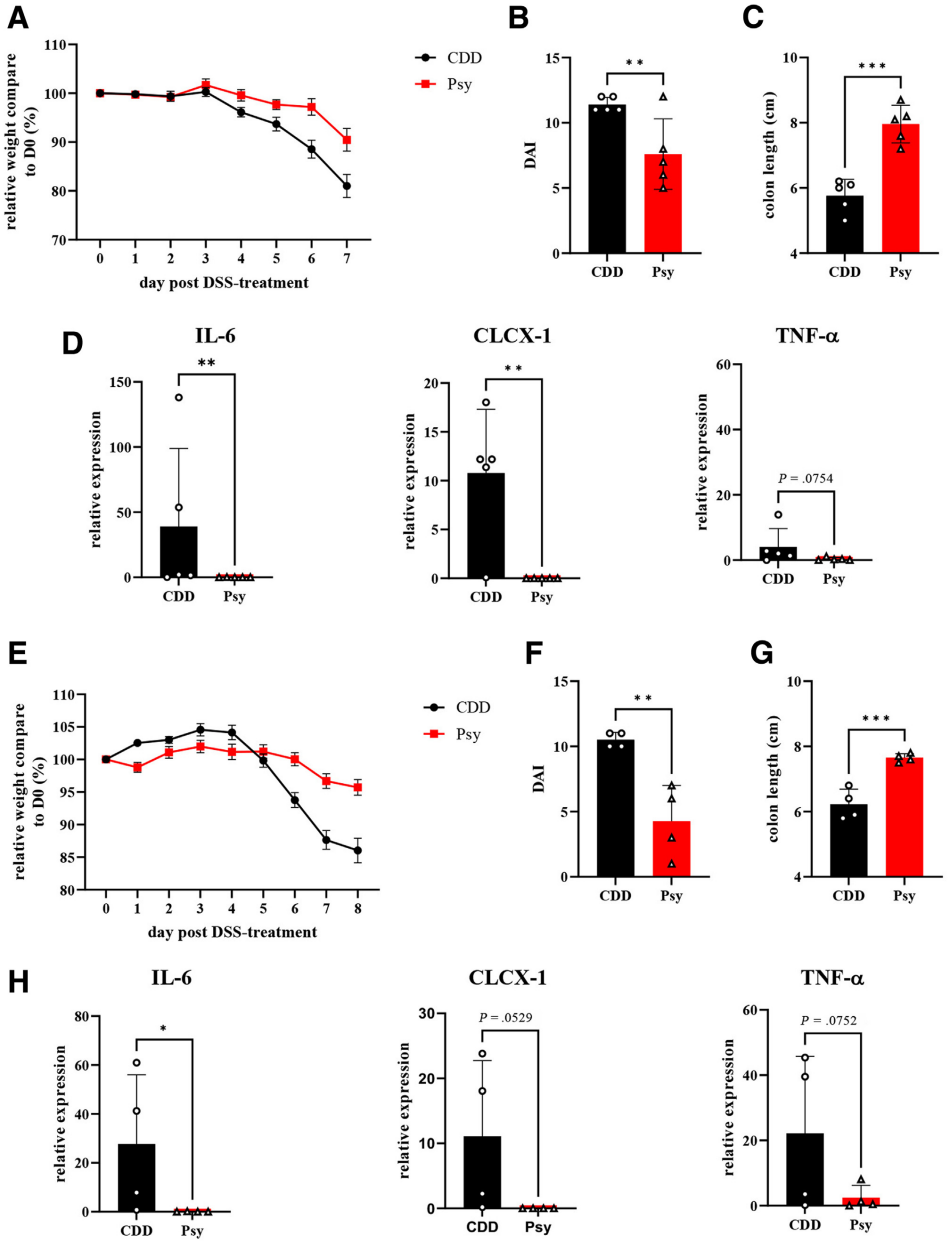
**FXR expression in neither epithelial cells nor hepatocytes was necessary for psyllium’s protection against DSS-induced colitis.** (*A–D*) FXR^Fl/FL^-Vil-CRE male mice were maintained on the specified diet for 7 days and subsequently treated with DSS 2.5% for 7 days. (*A*) Relative body weight over time. (*B*) Disease activity index (DAI). (*C*) Colon length. (*D*) Relative expression of pro-inflammatory cytokines. Data are expressed as means ± SD of n = 5 mice per group. This experiment was performed 2 times, each showing a similar pattern of results. (*E–H*) FXR^Fl/FL^-Alb-CRE male mice were maintained on the specified diet for 7 days and subsequently treated with DSS 2.5% for 8 days. (*E*) Relative body weight over time. (*F*) Disease activity index (DAI). (*G*) Colon length. (*H*) Relative expression of pro-inflammatory cytokines. Data are expressed as means ± SD of n = 4 mice per group. This experiment was performed 2 times, each showing a similar pattern of results. Significance was determined by Student *t* test except for panel *D*, where a Mann-Whitney test was performed. ∗*P* < .05; ∗∗∗*P* < .001; ∗∗∗∗*P* < .0001.

That administration of FXR agonists can protect against DSS colitis accords with other studies, but the cellular and molecular mechanisms by which it does so are unclear. Many FXR-induced genes, particularly in the liver, relate to BA production, whereas FXR activation suppresses BA biosynthesis. Yet FXR is expressed on a variety of cell types, and its activation also results in a variety of anti-inflammatory actions, including promoting production of anti-inflammatory cytokines, such as IL-10, and development of regulatory T-cells.^5^ These anti-inflammatory parameters can be readily envisioned to mediate psyllium’s suppression of T-cell mediated colitis and may also contribute to dampening inflammation in the DSS model. Studies focused on the DSS model observed that FXR activation results in inflammatory leukocytes being retained in the spleen, resulting in a reduced number of inflammatory phagocytes in the colon and, consequently, abrogation of disease severity. Such FXR-mediated attenuation of DSS colitis is associated with increased FXR expression specifically on dendritic cells (DC), suggesting a role for DC FXR. We are currently generating tissue-specific FXR-deficient mice in the hope of identifying the cell type on which FXR expression is critical for psyllium’s protection against colitis. To date, we have observed mice lacking FXR specifically in intestinal epithelial cells (FXR^Fl/FL^-Vil-CRE) or in hepatocytes (FXR^Fl/FL^-Alb-CRE) are both fully protected against DSS colitis by psyllium (Figure 13) and are currently generating mice to test the role of DC FXR in psyllium’s protection against colitis.

The observation that psyllium can protect against colitis is not new. Rather, psyllium also emerged as relatively unique in its ability to protect against DSS colitis in a screen of distinct fibers conducted by Faith and colleagues.9 Moreover, a range of human studies support that psyllium can benefit patients with colitis, with one study reporting psyllium to be as effective as the standard of pharmaceutical care, 5’-ASA, in maintaining remission.^26^

Relative to many studies of dietary fiber, psyllium’s beneficial actions against DSS colitis were observed at relatively low doses, including the lowest tested dose, which utilized a diet comprised of 2% psyllium by weight, resulting in mice consuming about 25 mg of psyllium per day. This equates to a dose of psyllium that would be difficult to achieve with supplements but could be achieved by consuming foods prepared with Plantago seeds. In any case, the concentrations used in our study are at least within an order of magnitude of the dose in published human studies, suggesting that the mechanisms in play may be similar. Aside from the role of FXR, our observations that psyllium impacts BA metabolism were made in non-colitic mice. Patients with IBD have reduced serum BA and reduced signaling by BA receptors, whereas effective treatment with anti-TNFα associates with restored serum BA. This suggests that, should psyllium impact BA metabolism in humans similarly to mice, such impacts might contribute to psyllium’s purported benefits to patients with IBD.

In addition to gleaning potential benefits of substances that may ameliorate disease and elucidating mechanisms of action, studies in animals also have potential to detect potential adverse effects. In this regard, we note that some mice fed the psyllium-enriched diets for prolonged periods exhibited mild elevations in aspartate transaminase and alanine transaminase. Such elevations were associated with very low levels of serum lipids. Neither elevated aspartate transaminase/alanine transaminase nor hypolipidemia was observed in mice fed high-fat psyllium diets. These results suggests that psyllium’s ability to chelate BA may make some hosts prone to become lipid-deficient, particularly if such hosts consume low-fat diets. Additionally, if high doses of psyllium, like high doses of inulin, might promote type 2 inflammation should be investigated. Lastly, we note that psyllium lowered microbiome alpha-diversity, which can often mark proneness to disease. These caveats notwithstanding, on the whole, our results accord with the view that psyllium consumption is generally beneficial. More specifically, our results support the notion that pharmacologic FXR activation might be useful in managing IBD, and thus, further investigation of its mechanisms of action are warranted. In particular, we suggest that future studies of psyllium in humans measure serum BA and consider roles for FXR activation in mediating impacts of this fiber.

## Methods

### Mice

C57BL/6 WT, Rag1^-/-^, and FXR^-/-^ mice were purchased from Jackson Laboratory (Bar Harbor, ME). Gnotobiotic mice with ASF were generated from C57BL/6 germ-free mice purchased from Taconic Biosciences Inc (Rensselaer, NY), as previously described.^27^ Tissue-specific FXR-deficient mice were provided by Dr Frank Gonzalez (National Institutes of Health, Bethesda, MD). All mice were bred, maintained, and experimentally studied at Georgia State University under an Institutional Animal Care and Use Committee (protocol # A17047).

### DSS-induced Colitis

Six- to eight-week-old male mice, which had been maintained on a standard GBC (Purina 5001) were, where indicated, switched to a purified “open-source,” herein referred to as CDD, the composition of which is indicated in Table 1, and fed ad libitum, on which they were maintained for the duration of the experiment. One week later, mice were administered drinking water containing 2.5% DSS (Lot # S7102, MP Biomedicals; MW: 36,000–50,000). DSS/water consumption was monitored every day. Mice were monitored daily for weight loss, stool consistency, fecal bleeding, occult blood, and until one of the groups (in most experiments, the CDD) had one or more mice reaching Institutional Animal Care and Use Committee endpoints (typically 7–9 days of DSS). Disease activity index was calculated by adding score from body weight loss compared with day of starting DSS treatment, stool consistency, and presence of blood in the stool (Table 2). Colon length, washed colon weight, and cecum and spleen weights were measured, and organs were collected for downstream analysis.

**Table 1:** CDDs Used in This Study

Product #	D12450J		D13081109		D18052507		D19021101		D19021102		D19021104		D19021103		D18052503		D19021105		D19021106	
	50 g Cellulose per 4057 kcals		200 g Cellulose per 4057 kcals		100 g Cell+100 g Glu per 4057 kcals		50 g Cell+150 g Inu per 4057 kcals		50 Cell g+150 g Pec per 4057 kcals		50 g Cell+150 g Hi-M per 4057 kcals		50 g Cell+150 g Psy per 4057 kcals		100 g Cell+100 g Psy per 4057 kcals		150 g Cell+50 g Psy per 4057 kcals		175 g Cell+25 g Psy per 4057 kcals	
	**gm%**	***kcal%***	**gm%**	***kcal%***	**gm%**	***kcal%***	**gm%**	***kcal%***	**gm%**	***kcal%***	**gm%**	***kcal%***	**gm%**	***kcal%***	**gm%**	***kcal%***	**gm%**	***kcal%***	**gm%**	***kcal%***
Protein	19	*20*	17	*20*	17	*20*	18	*20*	18	*20*	18	*20*	17	*20*	17	*20*	17	*20*	17	*20*
Carbohydrate	67	*70*	59	*70*	61	*70*	62	*70*	62	*70*	70	*70*	62	*70*	61	*70*	60	*70*	59	*70*
Fat	4	*10*	4	*10*	4	*10*	4	*10*	4	*10*	4	*10*	4	*10*	4	*10*	4	*10*	4	*10*
Total		*100*		*100*		*100*		*100*		*100*		*100*		*100*		*100*		*100*		*100*
kcal/gm	3.85		3.37		3.47		3.53		3.53		3.54		3.47		3.44		3.40		3.38	
Ingredient	**gm**	***kcal***	**gm**	***kcal***	**gm**	***kcal***	**gm**	***kcal***	**gm**	***kcal***	**gm**	***kcal***	**gm**	***kcal***	**gm**	***kcal***	**gm**	***kcal***	**gm**	***kcal***
Casein	200	*800*	200	*800*	200	*800*	200	*800*	200	*800*	200	*800*	200	*800*	200	*800*	200	*800*	200	*800*
L-Cystine	3	*12*	3	*12*	3	*12*	3	*12*	3	*12*	3	*12*	3	*12*	3	*12*	3	*12*	3	*12*
Corn Starch	506.2	*2025*	506.2	*2025*	468.7	*1875*	450	*1800*	450	*1800*	446	*1784*	468.7	*1875*	481.2	*1925*	493.7	*1975*	500	*2000*
Corn Starch, Hi-Maize 260	0	*0*	0	*0*	0	*0*	0	*0*	0	*0*	150	*240*	0	*0*	0	*0*	0	*0*	0	*0*
Maltodextrin 10	125	*500*	125	*500*	125	*500*	125	*500*	125	*500*	125	*500*	125	*500*	125	*500*	125	*500*	125	*500*
Sucrose	68.8	*275*	68.8	*275*	68.8	*275*	68.8	*275*	68.8	*275*	68.8	*275*	68.8	*275*	68.8	*275*	68.8	*275*	68.8	*275*
Cellulose, BW200	50	*0*	200	*0*	100	*0*	50	*0*	50	*0*	50	*0*	50	*0*	100	*0*	150	*0*	175	*0*
Inulin, Orafti HP	0	*0*	0	*0*	0	*0*	150	*225*	0	*0*	0	*0*	0	*0*	0	*0*	0	*0*	0	*0*
Pectin, Tic Gums 1400	0	*0*	0	*0*	0	*0*	0	*0*	150	*225*	0	*0*	0	*0*	0	*0*	0	*0*	0	*0*
Glucomannan	0	*0*	0	*0*	100	*150*	0	*0*	0	*0*	0	*0*	0	*0*	0	*0*	0	*0*	0	*0*
Psyllium	0	*0*	0	*0*	0	*0*	0	*0*	0	*0*	0	*0*	150	*150*	100	*100*	50	*50*	25	*25*
Lard	20	*180*	20	*180*	20	*180*	20	*180*	20	*180*	20	*180*	20	*180*	20	*180*	20	*180*	20	*180*
Soybean Oil	25	*225*	25	*225*	25	*225*	25	*225*	25	*225*	25	*225*	25	*225*	25	*225*	25	*225*	25	*225*
Mineral Mix S10026	10	*0*	10	*0*	10	*0*	10	*0*	10	*0*	10	*0*	10	*0*	10	*0*	10	*0*	10	*0*
Dicalcium Phosphate	13	*0*	13	*0*	13	*0*	13	*0*	13	*0*	13	*0*	13	*0*	13	*0*	13	*0*	13	*0*
Calcium Carbonate	5.5	*0*	5.5	*0*	5.5	*0*	5.5	*0*	5.5	*0*	5.5	*0*	5.5	*0*	5.5	*0*	5.5	*0*	5.5	*0*
Potassium Citrate, 1 H2O	16.5	*0*	16.5	*0*	16.5	*0*	16.5	*0*	16.5	*0*	16.5	*0*	16.5	*0*	16.5	*0*	16.5	*0*	16.5	*0*
Vitamin Mix V10001	10	*40*	10	*40*	10	*40*	10	*40*	10	*40*	10	*40*	10	*40*	10	*40*	10	*40*	10	*40*
Choline Bitartrate	2	*0*	2	*0*	2	*0*	2	*0*	2	*0*	2	*0*	2	*0*	2	*0*	2	*0*	2	*0*
Yellow Dye #5, FD&C	0.04	*0*	0	*0*	0.01	*0*	0.05	*0*	0	*0*	0.025	*0*	0.01	*0*	0.025	*0*	0	*0*	0.04	*0*
Red Dye #40, FD&C	0	*0*	0	*0*	0	*0*	0	*0*	0.05	*0*	0	*0*	0	*0*	0.025	*0*	0.01	*0*	0.01	*0*
Blue Dye #1, FD&C	0.01	*0*	0.05	*0*	0.04	*0*	0	*0*	0	*0*	0.025	*0*	0.04	*0*	0	*0*	0.04	*0*	0	*0*
Total	1055.05	*4057*	1205.05	*4057*	1167.55	*4057*	1148.85	*4057*	1148.85	*4057*	1144.85	*4056*	1167.55	*4057*	1180.05	*4057*	1192.55	*4057*	1198.85	*4057*
gm																				
Total Fiber	50		200		200		200		200		140		200		200		200		200	
Insoluble Fiber	50		200		118		50		50		50		147.5		165		182.5		191.25	
Soluble Fiber	0		0		80		150		150		0		39		26		13		6.5	
gm%																				
Total Fiber	4.7		16.6		17.1		17.4		17.4		12.2		17.1		16.9		16.8		16.7	
Insoluble Fiber	4.7		16.6		10.1		4.4		4.4		4.4		12.6		14.0		15.3		16.0	
Soluble Fiber	0		0		6.9		13.1		13.1		0.0		3.3		2.2		1.1		0.5	

Fiber contents listed are estimates based on these fiber sources containing 100% fiber and either 100% as soluble or insoluble.

Psyllium 92% total fiber, 26% insoluble, 65% soluble measured at an independent laboratory (Lee et al J AOAC Int 1992: 75(3): 395-416).

Glucomannan 97% total fiber with 18% insoluble and 80% soluble measured at an independent laboratory (Lee et al J AOAC Int 1992: 75(3): 395-416).

Hi-Maize 260 contains 1.6 kcal/g and is considered 60% fiber according to information on the technical sheet.

Inulin contains 1.5 kcal/gm as per Roberfroid, 1999 (J. Nutr. 129: 1436S–1437S, 1999).

Pectin and Glucomannan are highly fermentable don't have a specified kcal/g value, so we chose 1.5 kcal/g to match inulin.

Psyllium is considered 1 kcal/gm due to lower anticipated fermentability than pectin, glucomannan, or inulin.

**Table 2:** DAI Criteria

Symptom/score	Characteristics
Body weight loss	
0	No loss
1	1%–5% loss of body weight
2	5%–10% loss of body weight
3	10%–20% loss of body weight
4	>20% loss of body weight
Stool consistency	
0	Normal feces
1	Loose stool
2	Watery diarrhea
3	Slimy diarrhea, little blood
4	Severe watery diarrhea with blood
Blood in stool	
0	No blood
2	Presence of blood assessed by hemoccult II test
4	Visible bleeding

DIA, Disease activity index.

**Table 3:** List of Serum BA Species and Their Change Upon Psyllium Supplementation

Abbrevation	Common name	Class	Psyllium effect
CA	Cholic acid	Primary	Unchanged
CDCA	Chenodexycholic acid	Primary	Unchanged
DCA	Deoxycholic acid	Secondary	Decreased
GCA	Glycocholic acid	Primary, Glycine-conjugated	Unchanged
GCDCA	Glycochenodeoxycholic acid	Primary, Glycine-conjugated	Undetectable
GDCA	Glycodeoxycholic acid	Secondary, Glycine-conjugated	Undetectable
GHCA	Glycohyocholic acid	Primary, Glycine-conjugated	Undetectable
GHDCA	Glycohyodeoxycholic acid	Secondary, Glycine-conjugated	Undetectable
GLCA	Glycolithocholic acid	Secondary, Glycine-conjugated	Unchanged
GUDCA	Glycoursodeoxycholic acid	Secondary, Glycine-conjugated	Undetectable
HCA	Hyocholic acid	Primary	Unchanged
HDCA	Hyodeoxycholic acid	Secondary	Decreased
LCA	Lithocholic acid	Secondary	Unchanged
MCA(a)	a-Muricholic acid	Primary	Unchanged
MCA(b)	b-Muricholic acid	Primary	Increased
MCA(w)	w-Muricholic acid	Secondary	Decreased
TCA	Taurocholic acid	Primary, Taurine-conjugated	Increased
TCDCA	Taurochenodeoxycholic acid	Primary, Taurine-conjugated	Unchanged
TDCA	Taurodeoxycholic acid	Secondary, Taurine-conjugated	Decreased
THCA	Taurohyocholic acid	Primary, Taurine-conjugated	Undetectable
THDCA	Taurohyodeoxycholic acid	Secondary, Taurine-conjugated	Decreased
TLCA	Taurolithocholic acid	Secondary, Taurine-conjugated	Decreased
TMCA (a + b)	a- and b- Tauromuricholic acid	Primary, Taurine-conjugated	Increased
TUDCA	Tauroursodeoxycholic acid	Secondary, Taurine-conjugated	Increased
UDCA	Ursodeoxycholic acid	Secondary	Unchanged

BA, Bile acid.

### Adoptive T-cell Transfer-induced Colitis

Four- to six-week old Rag1^-/-^ male mice, which had been maintained on GBC, were, where indicated, switched to a CDD containing cellulose only or psyllium and cellulose indicated in Table 1, ad libitum, on which they were maintained for the duration of the experiment. One week later, mice were administered FACS-sorted CD4^+^CD25^-^CD44^-^ T cells (2 × 10^5^ per mouse, refer as CD45RB^high^) via intraperitoneal injection. Such T-cells had been isolated from spleens and mesenteric lymph nodes of WT mice via Naive CD4^+^ T Cell Isolation Kit (cat# 130-104-453) and CD25 MicroBead Kit (cat#130-091-072) of Miltenyi Biotec. Rag1^-/-^ recipient mice on indicated diets were monitored for clinical disease development (weight loss, diarrhea) and were euthanized when mice lost >20% of body weight compared with initial body weight.

### BA Quantification

Blood was collected by retro-orbital bleeding, and hemolysis-free serum was collected using serum separating tubes. Feces were collected, dried, and ground. Fifty mg of ground feces were placed into a glass tube. One mL of *t*-butanol:water (1/1) was added, then the tubes were vortexed and rotated at 37 ºC for 2 hours. Finally, samples were centrifuged at 800 *g* for 4 minutes at room temperature, and the supernatant was collected for analysis. Total BA were then measured in the serum and the supernatant from the feces using Mouse Total Bile Acids Assay Kit (Crystal Chem, Elk Grove Village, IL) as described by the kit. Quantitation of serum BA species was performed by the Michigan Regional Comprehensive Metabolomics Resource Core (Ann Arbor, MI).

### RNA Extraction and Real-time Polymerase Chain Reaction

Total RNA was isolated from colon using TRIzol (Invitrogen, Carlsbad, CA); the expression level of IL-6, CXCL1, and TNF-a was analyzed by using quantitative real-time polymerase chain reaction (PCR), according to the Biorad iScript One-Step RT-PCR Kit in a CFX96 apparatus (Bio-Rad, Hercules, CA) with the following primers (F/R): IL-6: GTGGCTAAGGACCAAGACC, GGTTTGCCGAGTAGACCTCA; CXCL1: TTGTGCGAAAAGAAGTGCAG, TACAAACACAGCCTCCCACA; TNF-a: CGAGTGACAAGCCTGTAGCC, CATGCCGTTGGCCAGGA; 36B4: TCCAGGCTTTGGGCATCA, CTTTATTCAGCTGCACATCACTCAGA. Differences in transcript levels were quantified by normalization of each amplicon to housekeeping gene 36B4.

### Histopathologic Analysis

Colons were fixed in 10% buffered formalin for 24 hours, embedded in paraffin, sectioned at 5-μm thickness, and stained with hematoxylin and eosin. Hematoxylin and eosin-stained slides were scored in a blinded manner by 2 pathologists (SW and DAW). Each colon was assigned 4 scores based on the degree of epithelial damage. For T-cell transfer colitis scoring, scoring was performed as described.^28^^,^^29^ In brief, 7 categories were scored: lamina propria inflammation (0–3), goblet cell loss (0–2), abnormal crypts (0–3), presence of crypt abscesses (0–1), mucosal erosion/ulceration (0–1), submucosal spread to transmural involvement (0–3), and number of neutrophils 40 objective magnification (0–4). The total histopathological score is then calculated by combining the scores for each of the 7 parameters for a maximum score of 17. For DSS colitis, colons were scored with a 23-point system in 8 categories as follows:^30^ inflammatory area (0–4), destruction of mucosal architecture (0–3), percent of lesion with mucosal ulceration (0–4), degree of cellular infiltrate (0–3), percent with severe transmural infiltrate (0–4), muscle thickening (0–3), crypt abscess (0–1), and goblet depletion (0–1).

### Bacterial Quantification in Feces and in Cecum Tissue

To measure the total fecal bacterial load, total DNA was isolated from weighted feces using QIAamp DNA Stool MiniKit (Qiagen, Hilden, Germany). DNA was then subjected to qPCR using QuantiFast SYBR Green PCR kit (Bio-Rad, Hercules, CA) with universal 16S rRNA primers 8F: 50-AGAGTTTGATCCTGGCTCAG-30 and 338R: 50-CTGCTGCCTCCCGTAGGAGT-30 to measure total bacteria number. Results are expressed as bacteria number per mg of stool using a standard curve.

### Quantification of Fecal Lcn-2 by Enzyme-linked Immunosorbent Assay

Freshly collected or frozen fecal samples were reconstituted in phosphate buffered saline containing 0.1% Tween 20 (100 mg/mL) and vortexed for 20 minutes to get a homogenous fecal suspension. These samples were then centrifuged for 10 minutes at 12,000 rpm and 4 °C. Clear supernatants were collected and stored at −20 °C until analysis. Lcn-2 levels were estimated in the supernatants using Duoset murine Lcn-2 ELISA kit (R&D Systems, Minneapolis, MN).

### Microbiota Sequencing

Mouse fecal samples were stored on dry ice or in −80 °C freezers until processing. DNA was extracted using the MagAttract PowerSoil DNA EP Kit (Qiagen). gDNA concentration was quantified using the Quant-iT PicoGreen dsDNA Assay Kit (ThermoFisher Scientific) on a BioTek Synergy H1 plate reader. DNA samples were then diluted to 0.375 ng/uL in molecular-biology grade TE buffer, and prepared for sequencing via Nextera library preparation as described by Karasov et al.^31^ Size-selection was carried out on a BluePippin (Sage Science) before sequencing on an Illumina HiSeq3000 with 2 × 150 nucleotide paired-end reads.

### Metagenomic Sequence Processing

Quality control of sequences was performed by first removing adapters and filtering raw sequences with bbtools v37.78 (bbduk with parameters fastawrap = 300 k = 23) and the software Skewer v0.2.2^32^ with the parameters of -n -l 100 -q 25. The bbmap command, part of the bbtools package (https://sourceforge.net/projects/bbmap/), was then used to remove any human reads potentially contaminating the samples (parameters minratio = 0.9 maxindel = 1 bwr = 0.16 bw = 12 fast minhits = 2 qtrim = r trimq = 10 untrim idtag printunmappedcount kfilter = 25 maxsites = 1). The fastqc software (v0.11.7) and MultiQC sofware (v1.5.dev0 88e1e4f) was used to generate summaries of final QC sequence counts. Reads passing quality control were then taxonomically profiled and their relative abundances estimated with Kracken2^33^ and Bracken (v2.2)^34^ using the Genome Taxonomy Database release 86 for the reference sequences and taxonomy.^35^

Raw Illumina paired-end sequence data are available in the European Bioinformatics Institute Sequence Read Archive under accession number PRJEB56541

### RNA Extraction, Sequencing, and Analyze

Total RNA from ileum was extracted using TRIzol Reagent (Thermofisher 15596026) and was further purified using QIAGEN RNeasy MinElute Cleanup Kit (Cat No./ID: 74204). The prepared RNA samples were then sent to Molecular Evolution Core of Georgia Institute of Technology for library preparation and sequencing on the NextSeq instrument utilizing a high output 2 × 75 bp run. The mouse reference sequence file (mm10) and the annotated general feature format (gff) file was downloaded from the USCS genome site (https://hgdownload.soe.ucsc.edu/downloads.html). The mouse genome index was constructed with bowtie-build in Bowtie. The fastq files were aligned to the reference genomic sequence by TopHat with default parameters. Bowtie2 and Samtools was used with the TopHat program. Estimation of transcript abundance was calculated, and the count values were normalized to the upper quartile of the fragments per kilobase of transcript per million fragments mapped reads using Cufflinks (cuffdiff). The processed data were then analyzed on software R for preparations of linear gene expression comparison, PCA, and heatmap figures. Heatmap figure represent the gene presenting a 2-fold increase and a *P* value < .05 between CDD and Psy diet. Webgestalt was used for the gene set enrichment analysis. A gene list for Webgestalt analysis was generated using the output from the cuffdiff program using the same set of gene than for the heatmap.

### Statistical Analyses

Statistical significances of results were analyzed using GrapahPad 9 software. Normality test was performed using the Shapiro-Wilk test. Experiments in which results showed normal distribution were tested by unpaired Student *t* test or analysis of variance. Experiments yielding data not following a normal distribution were analyzed by the Mann-Whitney test or Kruskal-Wallis test. When specified experiments ware performed multiple times, each showing a similar pattern of results, which mean that *P* values were similar between experiments (± 1 log_10_). Alpha and beta diversity were calculated using QIIME2.^36^ Bracken derived abundances were imported into QIIME2 and rarefied to 82502 sequences per sample. Shannon alpha diversity (with Kruskal-Wallis tests between diet groups) and Bray-Curtis dissimilarity were calculated using the diversity core-metrics plugin. The Shannon alpha diversity values were plotted using the R package ggplot2. Differences between the microbial taxonomic abundances of experimental groups were tested with LEfSe.^37^ The bracken OTU table was collapsed to the family level of taxonomy and rarefied to 82502 sequences per sample using QIIME2. The resulting table was then imported into the galaxy tool for LEfSe (https://huttenhower.sph.harvard.edu/galaxy/) for the 2 diets, CCD and psyllium, being compared. The default parameters were used for LEfSe, except for the threshold on the logarithmic LDA score, which was increased from 2.0 to 4.0. Significance is expressed as ∗*P* < .05, ∗∗*P* < .01, ∗∗∗*P* < .001, and ∗∗∗∗*P* < .0001.
